# Surface/Interface Engineering for High-Resolution Micro-/Nano-Photodetectors

**DOI:** 10.1007/s40820-025-01933-8

**Published:** 2026-01-03

**Authors:** Jinlin Chang, Ting Liu, Xiao Geng, Genting Dai, Liangliang Yang, Mingjun Cheng, Linpan Jiang, Zhenyuan Sun, Jianshe Liu, Wei Chen

**Affiliations:** 1 School of Integrated Circuits, Tsinghua University, Beijing, 100084 People’s Republic of China; 2 Beijing National Research Center for Information Science and Technology, Beijing, 100084 People’s Republic of China

**Keywords:** Photodetectors, Surface modification, High-resolution, Micro-/nanostructures

## Abstract

Photodetectors can convert light energy into electrical signals, so are widely used in photovoltaics, photon counting, monitoring, and imaging. Photodetectors are easy to prepare high-resolution photochips because of their small size unit integration. However, these photodetector units often exhibit poor photoelectric performance due to material defects and inadequate structures, which greatly limit the functions of devices. Designing modification strategies and micro-/nanostructures can compensate for defects, adjust the bandgap, and develop novel quantum structures, which consequently optimize photovoltaic units and revolutionize optoelectronic devices. Here, this paper aims to comprehensively elaborate on the surface/interface engineering scheme of micro-/nano-photodetectors. It starts from the fundamentals of photodetectors, such as principles, types, and parameters, and describes the influence of material selection, manufacturing techniques, and post-processing. Then, we analyse in detail the great influence of surface/interface engineering on the performance of photovoltaic devices, including surface/interface modification and micro-/nanostructural design. Finally, the applications and prospects of optoelectronic devices in various fields such as miniaturization of electronic devices, robotics, and human–computer interaction are shown.

## Highlights

- Surface/interface engineering can compensate for defects, adjust the bandgap, and develop novel quantum structures, which consequently optimize photovoltaic units and revolutionize optoelectronic devices.
- This review comprehensively elaborates on the surface/interface engineering scheme of micro-/nano-photodetectors from principles, types, and parameters, and describes the influence of material selection and manufacturing techniques.
- Surface/interface engineering continuously promotes the development of low-dimensional optoelectronic materials and drives the industrialization of flexible optoelectronic devices.

## Graphical abstract

**Figure:**
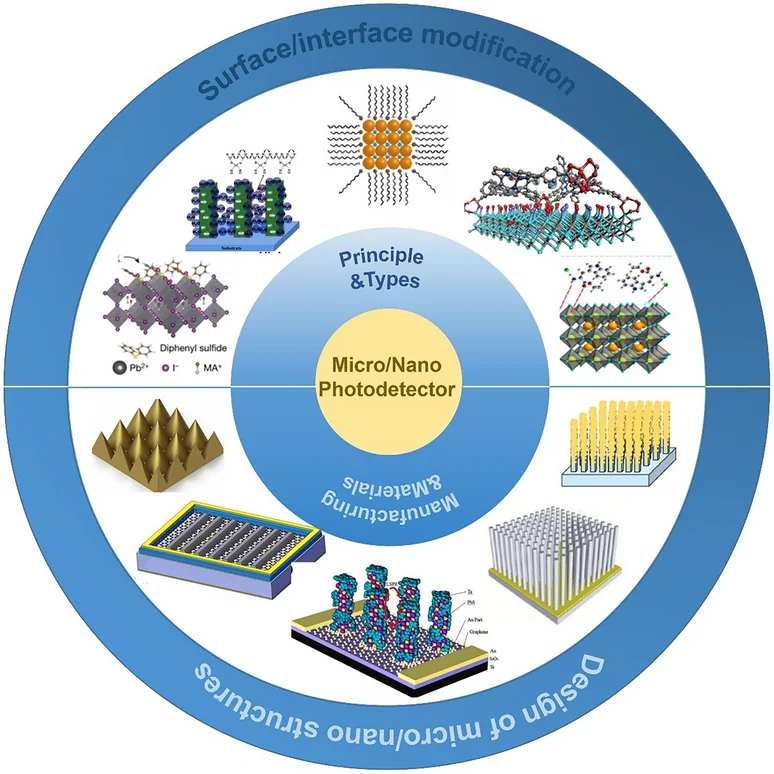

## Introduction

Light is one of the most important media for the transmission of information and energy, which is important for human interaction with the outside world. As a carrier, light transmits the contents of the environment to human beings, thus enabling them to establish an interactive system and make timely responses, which plays an irreplaceable role for humans. Recently, with the development of computers and artificial intelligence, light and artificial light detectors have gradually attracted widespread attention. Light detectors have similar functions to the human eyes and can convert external light information into electrical signals for computer demonstration and storage; they can also convert light energy into electrical energy as an energy converter. Therefore, the development of high-performance retinal-like photodetectors is of great significance for future artificial intelligence, human–computer interaction, and miniaturization of electronic devices.

Micro-/nano-optoelectronic devices are the main aims of photodetector development, which reduce the chip size by shrinking the optoelectronic unit while improving the signal information processing speed. However, the small size of the photodetector unit, especially the corresponding imaging unit, reduces the dynamic range and fill factor of the pixel, which is very unfavourable to the image output. Thus, the development of high-resolution sensors is also very important [1] (it is worth noting that in this paper, the resolution referred to is the lateral or longitudinal resolution based on the physical space, not the optical frequency/wavelength resolution based on the light wave). However, high resolution is very difficult to achieve because it implies more independent units per identical size of the macroscopic device, i.e. the smaller detector unit. This is usually a complex process involving precise regulation of material growth, accurate design of microstructures, innovations in photolithography, optimization of coating technology, and so on. Also, the performance of optoelectronic devices is greatly affected by defects and mismatches. Therefore, the realization of high-resolution micro-/nano-optoelectronic devices still remains a great challenge.

The small size characteristics of micro-/nano-optoelectronic devices are often based on the microscopic surface/interface; therefore, the development of surface/interface engineering is of critical significance for the optimization and innovation of the optoelectronic devices. Surface/interface engineering is mainly divided into two parts: one is surface/interface modification, mainly through chemical solvent treatment or functional group modification, so that the surface passivation or the formation of buffer layer, thus making the heterogeneous interface or the physical/chemical properties of the device surface undergoes a large transformation to modulate its light absorption or electron transport and improve the optoelectronic performance; it can also be achieved by branching or coating other substances to modulate the device's characteristics, such as the metal-induced localized plasma resonance. The second is the microstructure modulation at the surface/interface, or the setting of regular patterns with special functions, so the bandgap of the material or the function of the device is adjusted, so that the preparation of optoelectronic devices has high controllability. Wang et al. enhanced and modulated the localized plasmon resonance through the setting of metal elliptic-wall grating nanowires and thus improved the optoelectronic performance [2]. Ren et al. induced the formation of a PbI_2_ layer on the surface of CH_3_NH_3_PbI_3_ perovskite films by argon ion bombardment, which was able to effectively passivate defects and reduce carrier complexation in perovskite films, and improved the responsivity of optoelectronic devices by more than four times [3]. Photodetectors based on hollow nanorods were also capable of boosting the surface plasmon resonance and acting as a support, thereby enhancing the near-infrared wave response of metal-based devices by a factor of 60 [4]. Therefore, it is necessary to comprehensively analyse the surface/interface engineering of optoelectronic devices and their latest progress.

In recent years, significant progress has been made in surface/interface engineering for optical detection, breaking through device performance by precisely controlling material surface/interface characteristics such as band structure, defects, carrier transport/recombination, and light absorption. In 2004, the successful separation of graphene promoted the development of 2D electrons, leading to research on the correlation between 2D interfaces and electrical properties [5]. Based on this, building built-in electric fields to promote carrier separation by stacking low-dimensional materials or constructing van der Waals heterojunctions is the most popular interface engineering solution [6], including 2D MoS_2_/b-AsP/MoS_2_ heterojunction photodetectors [7], 2D ZnO/WSe_2_/graphene heterojunction photodetectors [8], 1D Bi_2_S_3_/2D WS_2_ heterojunction photodetectors [9], and 2D ReS_2_/0D MoS_2_ heterojunction photodetectors [10]. Nanoscale structures are also commonly used to enhance light absorption, including 1D nanowires, nanogratings, nanogaps, etc. Modifying passivation layers on the surface of materials to compensate for defects and suppress non-radiative recombination is also a common modification scheme. In 2009, Japanese scientists first used organic–inorganic hybrid perovskite materials to prepare the world's first perovskite solar cell device [11]. Perovskite, as an excellent photodetector material, is often optimized through multifunctional passivation due to the limitation of interface defects. In 2020, Zhao's team significantly improved the stability and performance of Sn Pb perovskite photodetectors through double-sided passivation, and expanded their visual applications [12]. Surface plasmon enhanced absorption has a long history, and traditional electromagnetic shielding absorbing materials are achieved through this effect. Metals with strong conductivity are more prone to this effect than other materials, and due to their easy preparation, they are currently widely used [13]. Flexible optoelectronic devices have great advantages in wearable applications, but they face the problem of organic/inorganic interface mismatch, resulting in transport losses, so optimizing the organic–inorganic interface is also very important. In 2020, Lei et al. reported a solution-based photolithography-assisted epitaxial growth and transfer method that can prepare single-crystal perovskite on any substrate, promoting the development of flexible optoelectronic devices [14]. In the emerging field of photodetector applications, surface and interface engineering plays a broader role. It can design a multifunctional interface with light detection, computation, and storage simultaneously. A deep understanding of the physical mechanisms of interfaces and the use of machine learning for multi-parameter collaborative optimization are an important aspect in promoting the industrialization of interface engineering [15, 16]. The development of interface engineering is crucial for breakthroughs in the performance of photodetectors.

Here, we introduce different types of optoelectronic devices from the principle of photovoltaic conversion and their evaluation parameters, which lay the foundation for the design and fabrication of high-resolution optoelectronic devices. After that, we introduce various photovoltaic materials, especially the emerging 2D materials, and show the advantages of each type of material, which provides guidance for the selection of materials for micro-/nanodevices. This paper also describes in detail the various optoelectronic device manufacturing processes and post-treatment programs and evaluates their applicability. Further, we introduce surface/interface engineering based on modification and microstructure design, showing its tremendous complementary and optimization features for materials and devices, and analyse its principle of action. Also, we present the application of photodetectors in many fields. Finally, we comprehensively analyse the current dilemmas and challenges faced by high-resolution micro-/nano-photodetectors, and propose solutions from the perspective of surface/interface engineering and look forward to the future of surface/interface engineering for optoelectronic devices.

## Photodetector Fundamentals

### Photodetector Principles

Early last century, based on Einstein's observation of the photoelectric effect and Planck's quantum hypothesis, scientists initially laid the cornerstone of the photodetector [17]. Over the past one hundred years, with the continuous development of solid-state physics, semiconductor technology and quantum technology, high performance, wide spectral range, large-scale integration of photodetectors mature products have played a key role in digital communications, measurement, microelectronic systems, low-power signalling systems and so on. They are almost ubiquitous in the work and life of modern people.

**Fig. 1:**
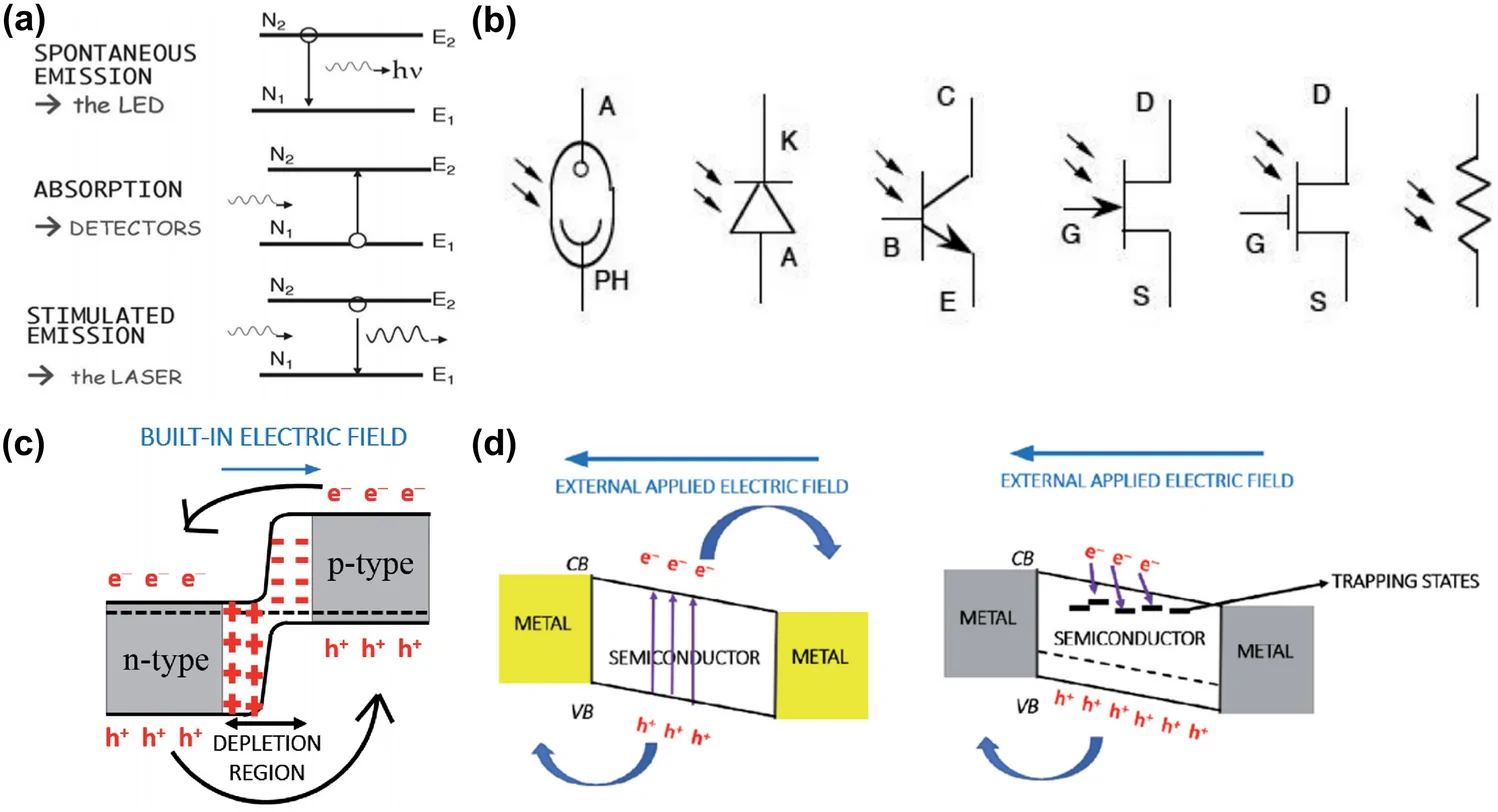
Principles and types of photodetectors. **a** Three types of interactions between external radiation and materials [17], Copyright 2021, John Wiley & Sons. **b** Circuit symbols of some photodetectors: phototube (or vacuum photodiode), photodiode, phototransistor, photo-FET, photo-MOS, and photoresistance [17], Copyright 2021, John Wiley & Sons. **c** Photovoltaic effect [18], Copyright 2021, BoD–Books on Demand. **d** Photoconductive (left) effect and photogating effect (right) [18], Copyright 2021, BoD–Books on Demand

There are three types of interactions between external radiation and materials: spontaneous emission, absorption and stimulated emission as shown in Fig. 1a. Among them, photodetectors are based on absorption. The radiation energy is quantized (hv), and during absorption, the energy of the radiation, hv, can only achieve absorption if it is greater than the energy level difference between the excited state and the ground state, also known as the threshold. The thickness of the material is also a key factor in achieving efficient absorption and conversion and to reduce losses [17].

Photoelectric effects are divided into two categories: external photoelectric effects and internal photoelectric effects [17, 18]. The external photoelectric effect refers to the photoelectric effect that can cause electrons to escape from the surface of an object under the action of light, also known as photoelectric emission. The external photoelectric effect is the physical basis of vacuum optoelectronic devices such as photocathodes and photomultiplier tubes. The difference between the internal and external photoelectric effects is that the incident photon does not directly bombard the photoelectrons from the inside of the optoelectronic material, but only excites the electrons inside the photoelectric material from the low-energy state to the high-energy state. The internal photoelectric effect includes the photoconductivity effect and the photovoltaic effect [17].

Photodetectors are electronic devices used to detect electromagnetic waves of different frequencies. When the photon energy is greater than or equal to the bandgap of the semiconductor material, the electrons jump to a higher energy level, forming hole–electron pairs, and the conductivity of the semiconductor material changes as a result. It quantifies the frequency and intensity information of the incident light through the conductivity changes of the semiconductor material, thus realizing light detection [18]. Based on the photoelectric effect, in which a photon or energy quantum is absorbed by materials and converted into an electron–hole pair, photodetectors can be divided into three categories, namely internal photoelectric devices, external photoelectric devices, and thermal detectors [17]. Internal photoelectric devices are photogenerated carriers that can form an external current loop, but do not produce excitation. When the energy is high enough, photoexcited electrons can be emitted from the material and thus collected or multiplied, which is the cause of external photoelectric devices (also known as photoemission devices). Because the emitted photoelectrons produce a higher signal intensity with less noise, they have great potential for distinguishing between device dark currents and single-photon detection; however, they have high bias voltage and narrow spectral range. Compared to them, internal photovoltaic devices have the advantages of wider applicability, good stability, wider spectral intervals, and the ability to be prepared in batches. Internal and external photodetectors are collectively referred to as quantum detectors [17]. In addition, thermal detectors are widely used photodetectors, which realize light detection by dissipating optical radiation in an absorber to generate heat and measuring it by a temperature sensor. It has a reduced sensitivity but an increased spectral range compared to photoelectric devices. Typical examples of photodetectors based on different mechanisms of action are shown in Table 1, and circuit symbols of some photodetectors are shown in Fig. 1b. Besides, photon resistance detectors, Golay cell, and photomagnetic detectors are some other forms of photodetectors which indirectly or by using weak interactions achieve light detection [17]. In this paper, we will mainly discuss internal photoelectric devices and thermal detectors. For internal photoelectric devices, the energy of the photon should be greater than the band gap for absorption to occur.

**Table 1:** Typical examples of photodetectors based on different mechanisms [17]

Number	Type	Examples
1	Photoemission devices (external photoelectric devices)	Vacuum photodiode, pickup tubes, gas photodiode, image intensifiers, photomultiplier and converters
2	Internal photoelectric devices	Semiconductor photodiode, CCDs (charge coupled devices), avalanche photodiode, SPAD (single-photon avalanche detectors), phototransistor (BJT: bipolar junction transistor, FET: field-effect transistor), photoresistance
3	Thermal detectors	Thermocouple (or photopile), thermistor (or bolometer), uncooled IR FPA, pyroelectric, IR vidicon

The essence of the photodetector is the absorption of quantized photons; then, carriers are excited to jump and obtain kinetic energy and migrate induced by the electric field within the device, generating a potential difference, thus converting light energy into electrical energy. From the principle, it can be informed that the detection of the light is quantized, as long as the sensitivity or precision of the material or device reaches a certain limit, single-photon detection is feasible, which requires upgrading of the photodetector or setting up specific operating conditions; from the principle, it is also possible to modify the material and design the microstructure, which leads to the adjustment of the bandgap and the precise manipulation of the working interval of the photodetector. The principle of photoelectric conversion plays a guiding role in the design of various types of photodetectors and also provides a target for each index of the device, and it is expected that a new type of optical device with high resolution can be developed from the principle, and its controllable design can be carried out.

### Photodetector Types

#### Photovoltaic Effect

##### Photodiodes

The photovoltaic effect refers to the effect of spontaneous generation of photocurrent in semiconductor devices under light. In semiconductor PN junctions, photogenerated electrons and holes are generated at the junction interface and separated by a built-in electric field, resulting in the directional generation of photogenerated currents, as shown in Fig. 1c [18]. In this type of photodetector, the detectable wavelength is limited by the type of semiconductor material, and the detectable wavelength can be adjusted by heterojunctions. Devices based on the photovoltaic effect require little external supply due to the presence of a built-in electric field and have a low dark current at zero bias, which is favourable to improve the performance of the detector [19]. Photodiodes are formed based on ordinary PN junctions with a reverse bias voltage applied. When illuminated, photons are absorbed and electrons are excited to jump to higher energy levels and can form hole–electron pairs. Meanwhile, the excess carriers in the space charge region are rapidly swept under the electric field, the electrons enter the N region, and the holes enter the P region, forming a transient photocurrent. The response speed of the diode is related to the carrier transport speed in the space charge region [20].

In order to generate larger transient photocurrents, PIN diodes with a wide space charge region have been designed. In a PIN diode, the N and P regions are separated by an intrinsic region, and the width of the intrinsic region is much wider than that of the space charge region of a normal diode, so that a larger space charge region is formed when a reverse bias voltage is applied, which consequently generates a stronger transient photocurrent.

Unlike homo-junctions, heterojunctions are constituted when the diode's nodal layer is composed of different semiconductors, i.e. materials with different energy gaps. The difference in the energy gap between the two sides of the heterojunction allows the photogenerated hole–electron pairs to be generally unabsorbed at the high-energy level and completely transparent, so finally arriving at the low-energy level side, which enhances the quantum efficiency and the response speed by reducing the dependence, showing excellent flexibility [17]. Another type of heterojunction is called Uni-travelling Carrier (UTC) Photodiode, which is created by inserting a thin P layer with a low-energy gap between the N region, the intrinsic region, and the P region, so that the light absorption in the limited frequency band is confined to the region of the thin layer and is rapidly collected with only little dissipation [17].

The Schottky photodiode is a photodiode based on a metal–semiconductor junction. Both the semiconductor and the metal can be used as the light detection section, but the metal layer is kept low to minimize reflection losses [17]. In this type of diode, the junction is very thin, which facilitates fast response. In addition, there are multispectral photodiodes, which form multiple junctions, each with a corresponding spectral absorption band, and thus multispectral light detection by growing materials with graded energy gaps directly in the high-energy and low-energy gap materials.

##### Avalanche Photodiode

**Fig. 2:**
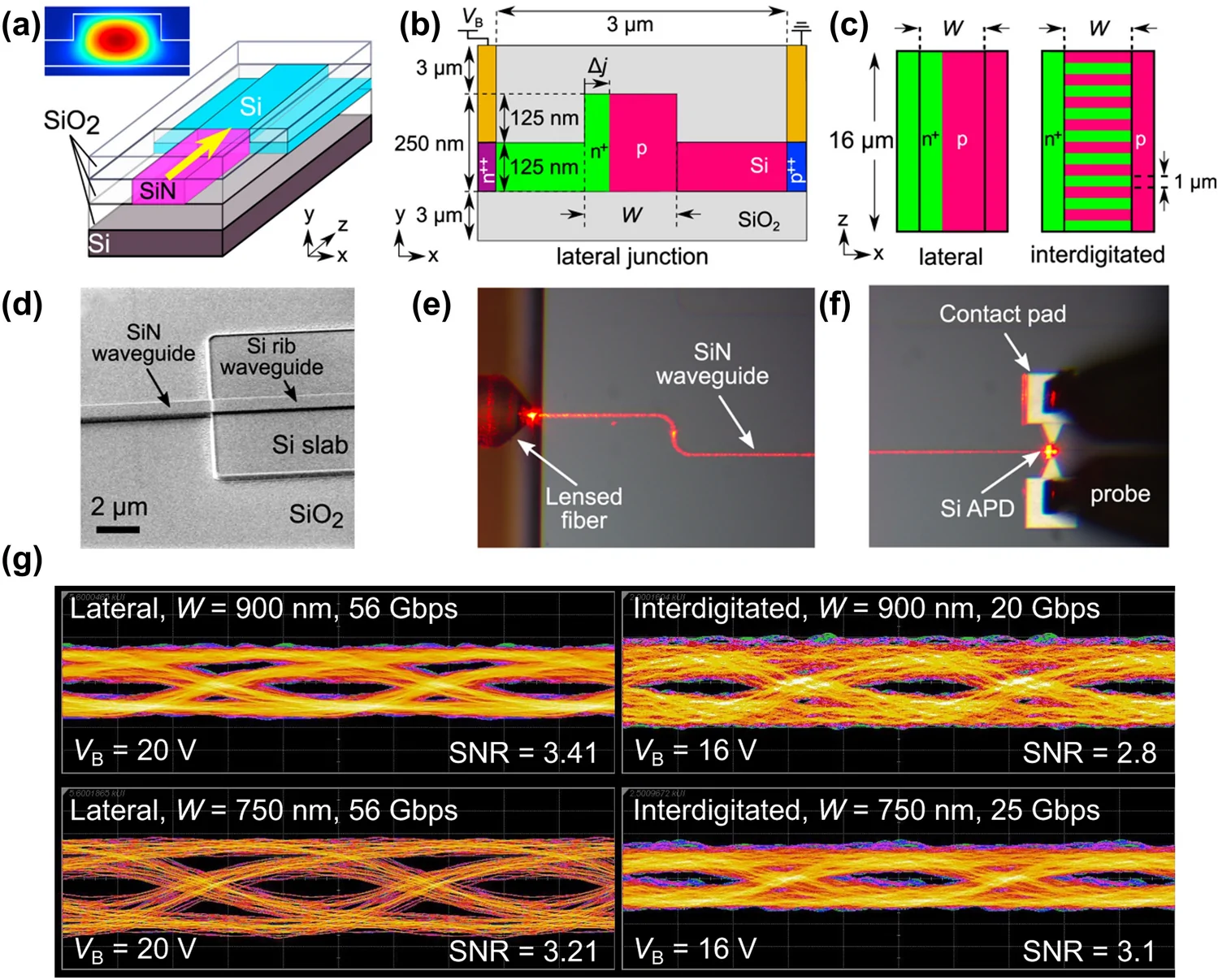
Integrated avalanche photodetectors [25], Copyright 2021, Nature Communications. **a** Schematic diagram of detector structure. **b** Detector cross-section. **c** Top view of detector, showing lateral (left) and interdigitated (right) doping, respectively. **d** Scanning electron microscope of detector. **e**, **f** Detector under an optical microscope, left: the lensed fibre coupling; right: Si avalanche photodetector regions; red line: due to the scattering of the 685-nm input light. **g** Measured eye diagrams for the lateral (left) and interdigitated (right) devices (SNR = signal-to-noise ratio)

Unlike ordinary photodiodes, avalanche photodiodes require a reverse bias voltage that is large enough to operate in the breakdown state and cause collisional ionization, which allows the photogenerated hole–electron pairs to continue to produce hole–electron pairs and boost the internal gain. The strong internal electric field promotes the photogenerated electrons and accelerates them to be able to ionize the lattice, generating new hole–electron pairs that are further accelerated and promoted for collisional ionization until they enter the cyclic circuit. When the space charge region reaches the electrode, punch-through of the junction occurs and a suitable operating voltage needs to be set to avoid perforation. However, since both holes and electrons move in the same direction, a positive feedback is created that attenuates the responsivity of the device and hence the need to maintain control of the proper avalanche gain [17]. Currently, a single-photon avalanche photodiode is being explored. Avalanche diodes are capable of responding to electromagnetic waves at microwave frequencies. Bartolo-Perez et al. have designed the photon trapping structure of a silicon single-photon avalanche detector to vary the depth of light penetration, thereby enhancing light absorption and boosting avalanche gain probability [21]. An integrated avalanche photodetector for the visible light is shown in Fig. 2.

##### Phototransistors

Unlike a photodiode, a transistor has three regions, the emitting region (E), the base region (B), and the collector region (C), which provides better stability and has the ability to control the flow of current with amplification [22]. The transistor can also be used as a photodetector. It forms a photocurrent by applying a reverse bias voltage to the B–C junction, and the larger B–C junction area facilitates the formation of a stronger photocurrent in the phototransistor. However, its larger junction area also results in a large increase in junction capacitance and a lower response frequency. Phototransistors include bipolar phototransistors, optocouplers, field-effect transistors (FETs), metal–oxide–semiconductor (MOS) FETs, and photoSCRs, etc. FETs are a commonly used photovoltaic device, which are voltage-controlled primitives with structural symmetry, large input resistance, and better stability than typical transistors [23].

#### Photoconductive Effect

##### Photoconductors

Photoconductors include some light-responsive semiconductor materials that produce excess carriers in the semiconductor when light is irradiated and the conductivity changes to enable light detection (photoconductive effect, Fig. 1d: left). However, these sensors usually require an external voltage to separate the photogenerated carriers and move them in a directional manner, which can have a large dark current and energy consumption [18]. Photoconductor detector devices have unrivalled advantages in small-signal measurements and new material development experiments due to their simple structure and easy control. Photoconductors have demonstrated excellent performance in industrial applications of single-element photodetectors, photometric devices, various types of infrared detection, imaging, etc. Cadmium sulphide and cadmium sulfo-selenide are common visible-light photoconductors; cadmium selenide is similar to cadmium sulphide but has a better conductivity to red light. Zinc sulphide can cover the ultraviolet band; lead oxide PbO and lead sulfo-oxide Pb (S, O) cover the near-infrared band; and doped Si, Ge, and gallium arsenide are also used as photoconductors. Currently, lower-cost organic photoconductors are also used in photodetectors, and they have shown great advantages in the field of flexible wearables.

The photogating effect is a special kind of photoconductive effect (Fig. 1d: right). When there are defects in the forbidden bands of semiconductors, the photogenerated carriers will be trapped in these regions, thus prolonging the carrier lifetime, suppressing carrier complexation, and enhancing the photovoltaic performance [24]. This phenomenon will be evident in low-dimensional materials with high specific surface area [18].

#### Photothermoelectric Effect

##### Photothermal Detector

**Fig. 3:**
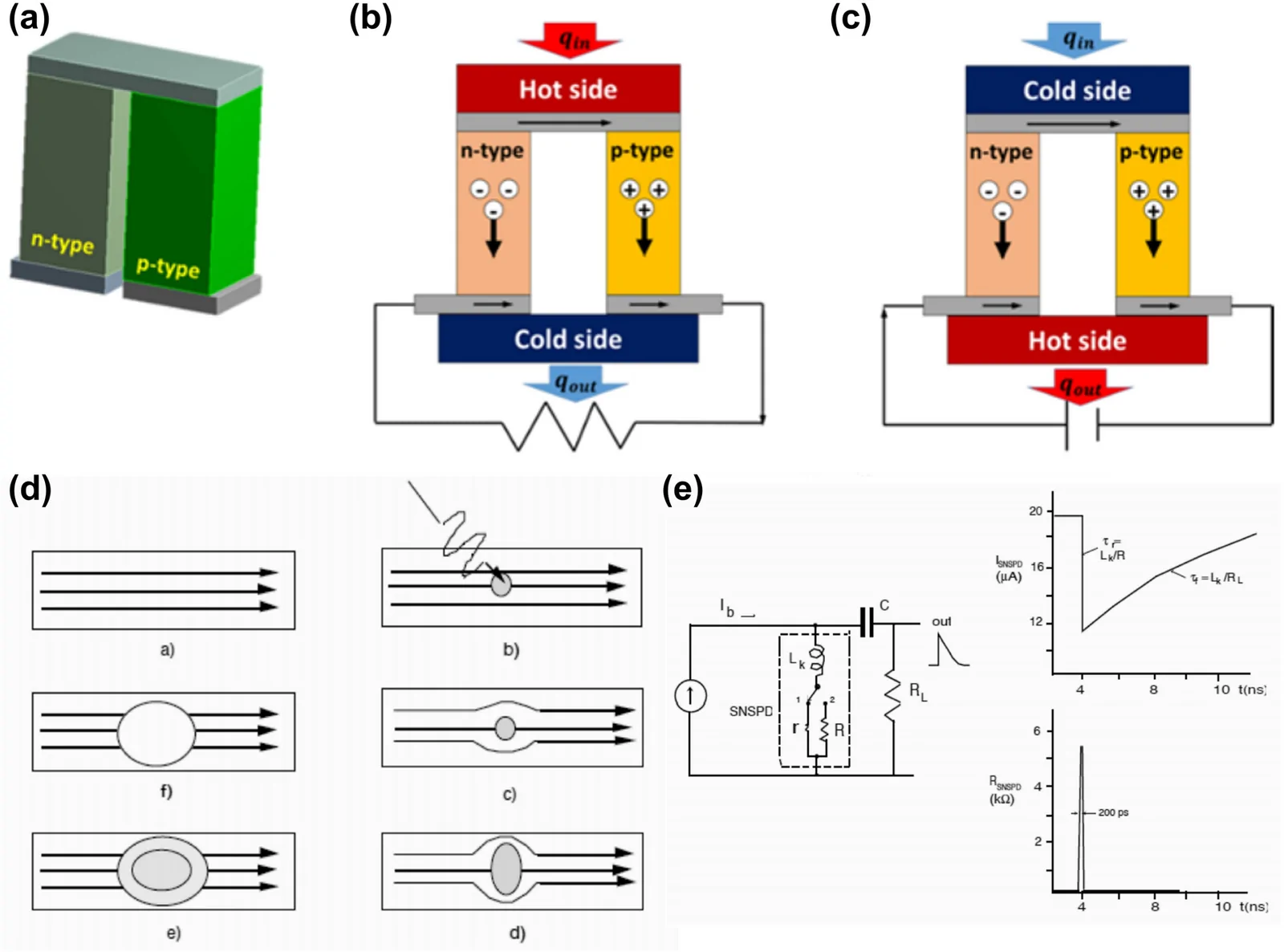
Thermal detector based on photothermal conversion. **a** Thermocouple. **b** Thermoelectric generation mechanism. **c** Thermoelectric refrigeration mechanism [28], Copyright 2018, Materials. **d** Operation process of superconducting photodetectors: (a) Superconducting state, (b) Photon injection, (c) Superconducting current transferring to the edge, (d) Hot spot formation, (e) Hot spot gradually occupies the entire width of the wire, (f) Generate pulses and restore to the initial state a). **e** Typical bias circuit of superconducting photodetectors and changes in detector current and resistance over time [17], Copyright 2021, John Wiley & Sons

Thermal detectors utilize the temperature change caused by light absorption, which is realized by a temperature detector. Compared with direct photodetectors, its indirect detection process will lead to a decrease in sensitivity and resolution, but it has a wider range of light detection interval [26], covering the ultraviolet to the far-infrared, and in the infrared band to show the cost advantage of quantum detectors are difficult to reach. It consists of two parts: an absorber (power dissipation section) and a temperature sensor. The absorber section is usually a very thin layer in direct contact with the probe of the temperature sensor. The absorber is usually black in colour and made of carbon black, precious metals, and other types of components, but the ultimate goal is to form a uniform photothermal conversion layer. In order to maintain stable photothermal conversion performance and improve accuracy, the coating process must be tightly controlled and maintain good adhesion to the substrate. There are typically three types of temperature sensors: thermocouples, thermistors, and pyroelectric sensors. Thermocouples (Fig. 3a–c) are based on a temperature difference to generate an electric current, which in turn detects heat; thermistors (also known as bolometers) are realized by changing resistance through temperature changes; and pyroelectric sensors are based on the special thermoelectric characteristics of pyroelectric materials (almost all piezoelectric materials), which are capable of generating a potential difference driven by a heat source. Thermocouple light detection does not require the driving of applied electrical energy, and it is extremely easy to realize self-powered detection devices; however, the limited amount of photogenerated heat, the lower potential of thermoelectricity, and the more energy conversion steps result in more energy consumption, which limits the application of this type of detector. A thermistor senses the amount of light by changing the resistance due to the heat generated in the radiation of uniform light [27]. In this process, the photosensitive material absorbs light energy and converts it into heat. The photothermal effect occurs mainly in the region of longer wavelengths such as infrared and requires external power support. Unlike thermocouples, pyroelectric effects depend on temperature fluctuations rather than gradients and are less controllable [28]. Temperature changes can cause spontaneous polarization of some materials, resulting in pyroelectric effects [29]. Pyroelectric materials are usually connected to two conducting electrodes to form a capacitor, which enables charging and discharging [30]. Having a nonzero dipole moment, no centre of symmetry and the absence of an axis of rotational symmetry or the absence of a unique axis of rotational symmetry are the basic characteristics of pyroelectric materials [30]. The pyroelectricity and piezoelectricity of a material are somewhat related and influence the physical application of the material by acting on the electrical, thermal, and mechanical characteristics; pyroelectric materials are also a type of piezoelectric material [31].

MEMS-based optical power meters are usually realized with the help of the photothermal effect, which can reflect the optical power by bending the cantilever beam through the difference in thermal expansion coefficients of the bimaterials [32], or by shifting the resonance frequency and so on through the ultrasensitive heat-absorbing property of the resonant cavity, which leads to the broadband optical power measurement [26].

##### Superconducting Detector

Superconducting detector is a photothermal detector. When the temperature is below a critical value (typically 1–10 K), many materials exhibit superconductivity, with resistance dropping to zero and no scattering losses. At this time, electrons are bound in Cooper's pairs, and the bond is broken when the temperature exceeds the critical temperature. The superconducting state is also broken when the current density is greater than a critical value. The kinetic inductance of the device prevents this phenomenon, causing a pulse in the circuit. Indeed, the kinetic inductance is specific to high-mobility materials like the superconductor [17]. The superconducting light detector is realized by using this phenomenon; the process is shown in Fig. 3d. The pulse waveform can be explored by means of the circuit shown in Fig. 3e: the circuit is biased by a constant-current generator, and the pulse is acquired by the load RL and coupled to the superconducting detector. When a photon is detected, the resistance of the superconducting detector bursts from r to R. However, due to the presence of the inductor, the current does not burst immediately and a voltage VL equal to the voltage drop across R is generated at the resistor switch, after which the inductor interacts with R and the current decreases. Also, due to the presence of the constant-current bias, the pulses will be very fast. As the current decreases and heat enters the substrate, the device returns to the superconducting state and the inductance gradually recovers with excellent timing performance [17]. Superconducting detectors automatically close the absorption of other photons when detecting a single photon and the pulse is unaffected by other photons, i.e. without photon resolution. Single nanowires have a very small cross-section that focuses the signal and obtains optimal efficiency [17]. The absorption efficiency can be increased by placing the nanowire in a resonant cavity. Single-mode fibres can be used to connect to the device from low temperatures to room temperatures.

#### Other Effects for Photodetector

##### Piezophototronic Effect

Piezoelectric materials can also be used to control carrier generation and complexation through pressure and can improve carrier lifetime in photodetectors to enhance photodetection [27]. Piezoelectric electronic devices are usually based on PN and Schottky nodes, which improve carrier transport at the junctions by introducing piezoelectric ionic charges. The introduction of the pressure-induced piezoelectric effect leads to a lowering of the junction barrier and carriers can pass through the junction more easily with enhanced responsivity, while the opposite situations occur for the tension-induced piezoelectric effect [33].

##### Bulk Photovoltaic Effect

Bulk photovoltaic effect is a nonlinear phenomenon of excitation light polarization controlled by the quantum geometry in optical leaps [34, 35]. The effect exists in arbitrary non-centrosymmetric systems and depends only on their Bloch states and energy bands, the efficiency is not constrained by the Shockley–Queisser limit, and the semiconductor bandgap does not affect the open-circuit voltage. Ferroelectric systems, twisted double bilayer graphene, and others have demonstrated feasibility in optoelectronic detection, which is of great significance for the micronanal integration of photodetectors and reduction of the chip size [34, 36].

Different types of photodetectors have their advantages and disadvantages. The traditional diode, transistor because of its low cost, mature process, stability, reliability and other advantages still occupy a major market. However, other types of new photodetectors have outstanding advantages, which enable them to replace traditional devices and continue to develop. Avalanche diodes operate in the breakdown state with higher internal gain, thus improving the photoelectric conversion. Superconducting photodetectors are capable of single-photon detection and can detect very low-energy photons, but their operating environment needs to be controlled at very low temperatures. There are several types of photothermoelectric detection systems, depending on temperature difference, thermistors and pyroelectric materials. Compared to direct photoelectric conversion, photothermal conversion tends to have more energy loss, but its efficiency is relatively high because it does not require photoexcited electron leaps, and even longer wavelength, lower energy infrared light can be absorbed by the conversion, with a wider absorption band. Piezoelectric materials can also act on optoelectronic devices by optimizing carrier complexes and so on. In recent years, with the development of 2D materials, the body photovoltaic effect also provides new ideas for the development of new photodetectors. The development of photovoltaic devices has been advancing in the process of continuous exploration and clarification of the principles; new materials and new effects are also very important for the design of photovoltaic devices, and the future development and innovation of photovoltaic devices should be based on the discovery of new phenomena and exploration of new principles.

### Photodetector Parameters

In order to better compare the performance of different photodetectors, it is necessary to contrast the parameters of the devices, so FIGURES OF MERIT OF DETECTORS is very important. In the photoelectric conversion process, the absorbed light energy cannot all be converted into electricity, there are some losses; therefore, the photoelectric conversion efficiency, i.e. quantum efficiency is a key indicator, that is, the average ratio of electrons to absorbed photons. The higher the quantum efficiency, the lower the losses. Photon loss and surface loss occur near the threshold, so the photoelectric conversion efficiency of a material gradually deviates from the theoretical value as it approaches the threshold. Related to this, the ratio of the output current density to the absorbed light density is the spectral sensitivity. The longer the wavelength, the greater the number of photons at the same energy, so the higher the spectral sensitivity [17].

Photodetectors are affected by noise while outputting a signal. There are two main sources of noise that are independent of each other: one is thermal noise (aka Johnson) originating from the load resistor; the other is quantum noise (aka shot noise) originating from the signal current and dark current. Both bandwidth and noise are key factors to be considered in the optimization of photodetection performance and can be appropriately modulated by the load resistance. A small load resistance is beneficial to increase the bandwidth of the detector, while a large load resistance can reduce the total noise fluctuation to improve the sensitivity, so choosing the appropriate load resistance, designing the optimized equalization circuit structure, and providing the device gain are the important contents of optimizing the optical detector [17]. It is important to note that sensitivity is the ability to detect small signals rather than signal detection quantities.

The two types of noise have variable weightings in different situations. When the quantum noise is greater than the thermal noise, especially when the signal current is much smaller than the dark current, the photodetector recognizes smaller photon absorptions with higher sensitivity, which means that the load resistance is on a higher order of magnitude, usually up to the level of G ohms [17]. There exists a critical signal current (also known as the threshold of quantum regime), when the signal current is greater than the threshold value, the signal-to-noise ratio (S/N) reaches the quantum noise limit of detection, following the Poisson photon statistic, which has also been called quantum regime. The lower the critical signal current, the higher the sensitivity of the detector. Correspondingly, when the signal current is less than the critical signal current, the noise is mainly contributed by the load resistance, which is also known as the thermal regime of detection. Many semiconductors, artificial superlattices, quantum wells, and other materials with a targeted bandgap are used for optical detection [37]. Lower dark current and higher quantum efficiency are favourable to enhance the S/N of the detector.

The noise equivalent power (NEP) is the ratio of the output noise to the responsivity, and the performance of the detector is inversely proportional to the NEP, thus defining the detectivity (D) as the reciprocal of the NEP. D is related to the detector's parameters and is affected by the detector's area (A) and bandwidth (B), thus defining the detector's detectivity [17]:

1 $$D^{*} = D\sqrt {AB}$$

which is also known as specific detectivity [18].

Background-limited-intrinsic-performance (BLIP limit) is also an important physical parameter for photodetectors, and D(BLIP) shows the detection rate limit on a thermal background. D(BLIP) is dominated by thermal noise, which has a minimal effect in the visible and near-infrared. It works with D* at different noise weights, respectively. In single-photon detection, the noise is mainly affected by the quantum-efficient dark current [17].

In photodetectors, the end-state outcome is present in the output current, and therefore, the output photocurrent is a fundamental index [18]:

2 $$I_{p} = I_{{{\mathrm{Illuminated}}}} - I_{{{\mathrm{dark}}}}$$

*I*_Illuminated_ is the output current produced by the device when illuminated; *I*_dark_ is the output current of the device when not illuminated.

*Responsivity* is then the ratio of the photocurrent to the incident optical power in the corresponding frequency band [19],

3 $$R_{\lambda } = \frac{{I_{p} }}{P \times A}$$

where *P* is the incident light power density and *A* is the light area of the device. A higher response rate indicates that more of the light energy is converted into electrical energy and better detection is achieved.

The internal gain (G) is the number of electrons that can be collected at the electrode by a single photon incidence [18, 38]:

4 $$G = \frac{{hc*R_{{\gimel }} }}{{\eta e{\gimel }}} = \frac{\tau }{t}$$

h is Planck's constant, c is the speed of light in a vacuum, ℷ is the wavelength of the incident light, η is the external quantum efficiency (EQE) of the device, and $R_{{\gimel }}$ is the responsivity of the device. G is essentially the ratio of the hole carrier lifetime (τ) to the electron transfer time (t) [39]. In order to increase G, we can capture carriers to prolong the carrier lifetime.

The sensitivity of a detector is the ratio of photocurrent to dark current, and the sensitivity of a device can be improved by either boosting the photocurrent or lowering the dark current. Typically, defect-free, high-quality devices favour higher photocurrents [18].

*Response/recovery time*: For photodetectors, the speed of detection of incident light and the speed of recovery to the initial state after the detection is complete are also key metrics; the faster these speeds are, the faster the response of the sensor is, and they are largely controlled by the junction [18]. In addition, series load resistance, junction capacitance, etc., also have an effect on response/recovery time.

Typically, photodetectors require an external power supply to drive and hold the photogenerated electron holes, so external energy consumption is also a part of the device design that needs to be taken into account. Self-supply, zero-bias, etc., are promising ways to develop energy control [40, 41].

*Spectral range*: Typically, photodetectors can be categorized into three types: broadband, narrowband, and very narrowband (i.e. wavelength selective) [18]. Broadband usually consists of heterojunctions. Wavelength-selective photodetectors are of two main types: one uses a single semiconductor as the active material (single bandgap); the other reflects electromagnetic waves of a specific wavelength multiple times with the help of an optical microcavity (Distributed Bragg Reflector (DBR)) and enhances the photocurrent [18]. Wavelength selectivity can also be achieved in broadband spectral detection with the help of polar switching phenomena [42].

Standardized parameters are essential for the evaluation of optoelectronic device performance; they not only reflect device specifications and provide guidance for device optimization, but also make it easier to compare various types of photodetectors laterally, thus facilitating communication between researchers, companies, and different regions. Moreover, many parameters are based on the device, but the index of material photoelectric performance is usually related to the volume, area, density and other physical quantities of the material, so the comparison of photoelectric parameters based on devices of different sizes is inappropriate, and the development of parameters based on the limited physical quantities is also very valuable. Through these parameter values, we can more easily determine the advantages and shortcomings of the device, thus providing guidance for the upgrading and optimization of high-resolution micro-/nano-optoelectronic devices. Some other photodetector parameters are shown in Table 2 [17].

**Table 2:** Some other photodetector parameters

Number	Parameter	Meaning
1	Spectral sensitivity	(σ): Detection of the relative efficiency of light or other signals as a function of the frequency or wavelength of the signal; by this parameter, the optimal optoelectronic material can be selected within the target wave frequency band
2	Active area	Photosensitive area; proportional to the reverse current, the junction capacitance and the cost
3	Dark current	The current flowing in optoelectronic devices without light irradiation; improves as the thresholds of the device and depend on Temperature
4	Barrier capacitance	Equivalent capacitance of depletion layer width variation; related to junction area, depletion layer width, dielectric constant of semiconductor, and applied voltage
5	Bias voltage	i.e. Vbb: the DC potential of the base to ground of a transistor under static bias conditions
6	Series and parallel resistances	Series resistance = substrate resistance + lateral resistance + electrode resistance + contact resistance; the series resistance and the junction capacitance both reduce with increasing Vbb. The parallel resistance reflects the leakage level of the device; affects the open circuit voltage, but has little effect on the short circuit current
7	Response uniformity	Decreases by 5%-10% near the edge of the active area; also affected by the incident angle
8	Temperature coefficient	= dσ/σdT. The temperature coefficient curve is zero at the peak of quantum efficiency, and if the wavelength is less than the peak, the temperature coefficient is negative, otherwise it is positive
9	Temperature range	Normal operating temperature range of the device; includes normal temperature range and the extended range
10	Reproducibility of the characteristics	For identical devices, σ at the peak of quantum efficiency is ± 1%, dark currents are ± 50%, temperature coefficient is ± 0.1%/°C
11	Reliability and MTTF (mean time to failure)	Comparable for small-signal diode due to the similar technology, MTTF usually 3–10*10^7^ h
12	Electrostatic damage	Ternary photodiodes (InGaAs and GaAsP) are easily damaged by electrostatic discharge
13	Ambient performances	Ambient-stress tests by Standards Committees

## Material Choice and Manufacturing

### Material Choice

Silicon-based photodetectors are the most classical and widely used type, and silicon solar cells have been put into widespread industrial applications. However, the energy generation gap of silicon limits it to visible light detection only, and it is powerless for the infrared band, which can be improved by integrating germanium on its surface [18]. Currently, with the discovery and development of new materials, many substances with excellent optoelectronic properties are used in optoelectronic devices, such as 2D materials, perovskites, and transition-metal dichalcogenides (TMDCs).

#### Black Phosphorous

As an emerging material, 2D materials have great potential for the preparation of high-performance nanoelectronic devices. In photodetection, the small size of 2D materials facilitates the reduction of dark current, but the ultra-thin thickness also leads to low light absorption and low quantum efficiency, and it is important to explore 2D materials and optimize their S/N for photodetectors [37, 43]. Reducing the preparation cost of 2D materials and simplifying the processing flow are important for the application of 2D photodetectors.

**Fig. 4:**
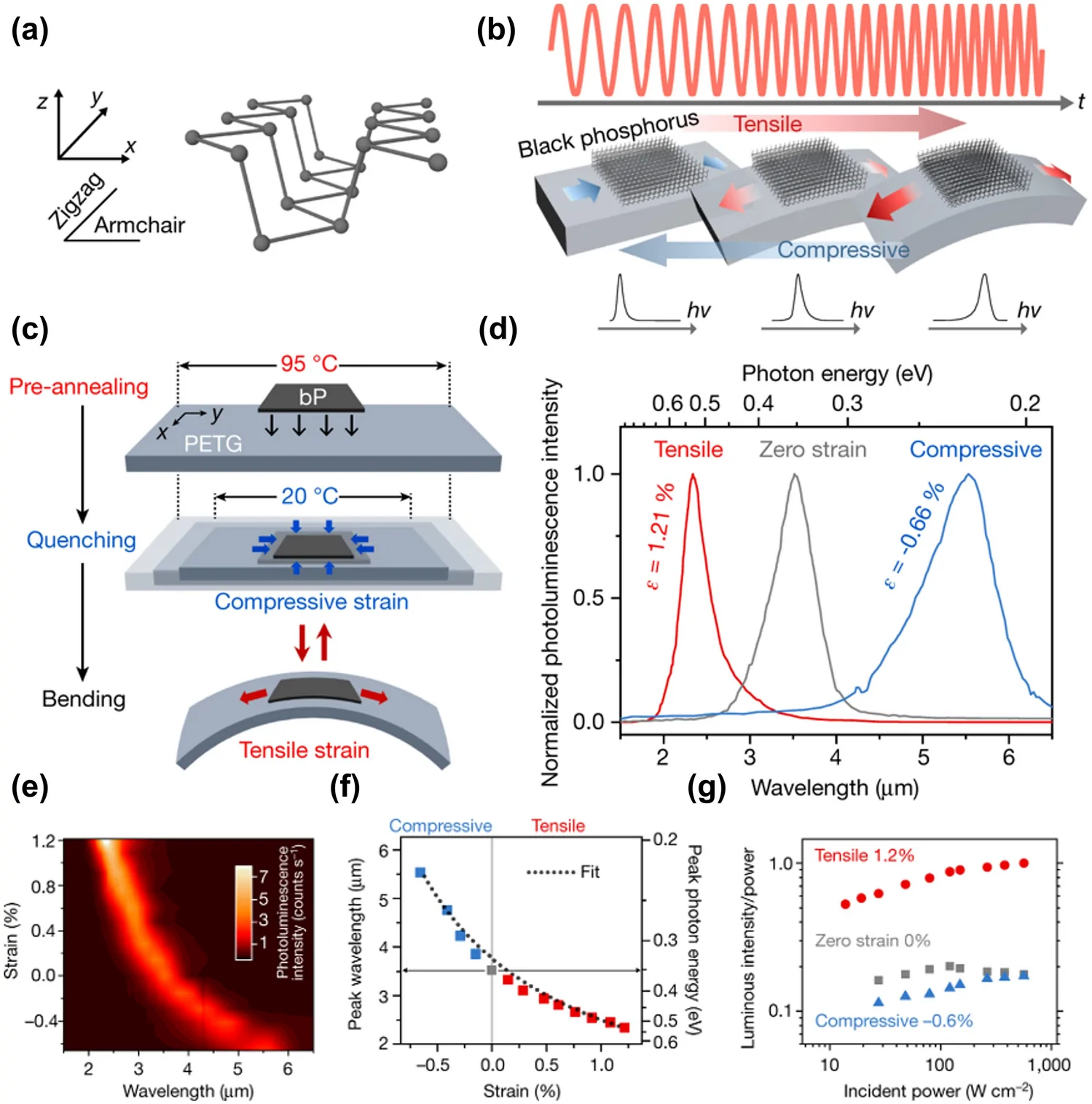
2D black phosphorus photodetector [52], Copyright 2021, Nature. **a** Crystal structure of black phosphorus. **b** Tunable bandgap of black phosphorus induced by strain. **c** Strain demonstration. **d** Normalized photoluminescence spectra of black phosphorus under different strains. **e** Photoluminescence spectra of black phosphorus. **f** Strain-dependent shift of photoluminescence spectral peak in black phosphorus. **g** Integrated photoluminescence intensity normalized

2D black phosphorus, a 2D material just discovered in 2014, has high hole mobility and anisotropy, which makes it an ideal optoelectronic material. Moreover, it can be designed in different layer stacks with van der Waals force interactions between the layers, is sensitive to doping and optical polarization, and has a narrow bandgap that enables detection in the near infrared [44–46]. However, low optical absorption limits its quantum efficiency [47]. Long et al. achieved effective room-temperature infrared detection and extended the detection wavelength by as doping [48]. Black phosphorus carbides also exhibit broader absorption spectra and mobility [49]. Their anisotropy also facilitates optical polarization detection. Its controllable thickness also facilitates dark current tuning, which enhances S/N [50]. Engel et al. prepared a multilayer black phosphorus photodetector capable of operating in the visible and infrared and obtaining high-resolution, diffraction-limited images [51]. Conventional photodetectors achieve operating wavelength tuning through heterogeneous structures with component coordination during material growth. However, the fabrication of such devices is complex and the range of adjustment after moulding is limited, so the development of band-tunable photodetectors is of great significance. The bandgap of black phosphorus has a high strain sensitivity (0.22–0.53 ev), thus enabling reversible, continuous, band-tunable photodetection, as shown in Fig. 4 [52].

#### Graphene

Graphene is a good absorber that can absorb electromagnetic waves in a wide range of frequency intervals. However, its low absorption efficiency and high susceptibility of carriers require further improvement for practical applications. By designing quantum dot-like structures or integrating them with quantum dots, graphene is able to better trap and transport carriers and improve the device's ground responsivity [53, 54]. Quantum dot-based photodetectors often require special methods to optimize the functionality and durability of quantum dot structures, but such treatments are incompatible with conventional semiconductor device preparation processes. Ahn et al. were able to protect quantum dots with the help of conventional photolithography and dry-etching of graphene monolayers to avoid complex and incompatible treatments [55]. Liu et al. also improved carrier lifetime by designing a tunnelling layer between the graphene layers to enhance the response rate [56]. However, both of these methods slowed down the carrier collection efficiency, resulting in slow responsivity, which can be improved by using metallic antenna nanoarrays [57]. Currently, the use of 2D graphene combined with a single-crystal silicon substrate to replace the metal in conventional Schottky nodes is a promising direction [18].

Graphene's excellent light absorption and photothermal conversion performance also facilitate light detection through thermal detection. Good homogeneity and weakening of external interactions are key to improving the performance of thermal detectors. Thermal insulation structures can optimize photothermal conversion and reduce external effects [58]. The absorption bands of graphene photodetectors can also be tuned, which is achieved by plasmons in graphene manipulated by gate voltage [59].

Similar to graphene, there is also a silicene 2D material that has a similar hexagonal structure to graphene, replacing carbon with silicon. The photovoltaic conversion properties of silicene are similar to those of graphene; however, its silicon-based qualities favour its combination with the currently mature silicon chip technology, which has very promising applications [18].

#### Perovskite

**Fig. 5:**
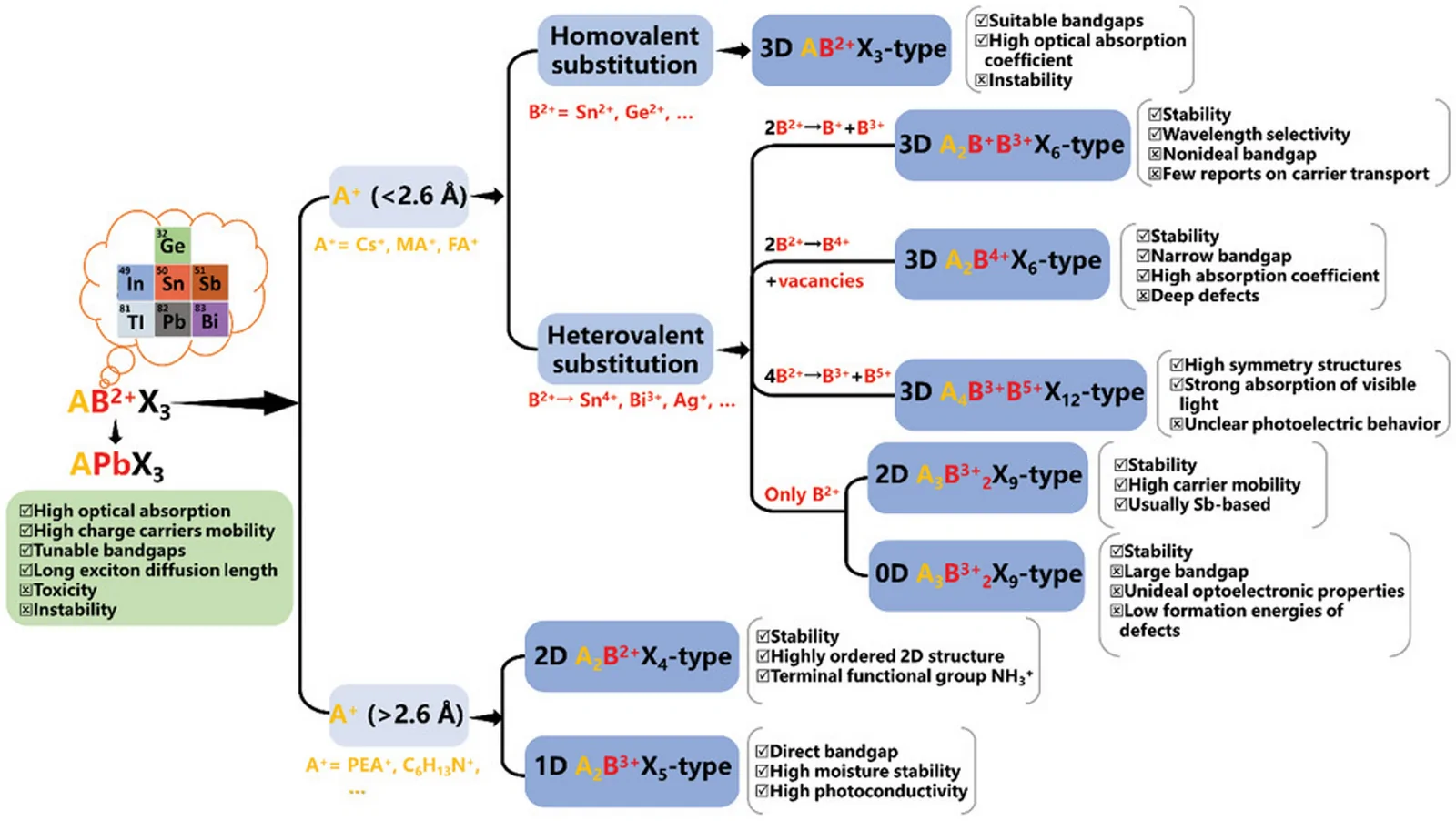
Two strategies for the formulation of the lead-free perovskite materials [65]

Perovskites generally have an ABX_3_ structure, where A is an organic cation (CH_3_NH_3_^+^ or MA^+^, FA^+^), B is inorganic cations (Pb^+2+^ or Sn^2+^, Ge^2+^), and X is halide anions (I^–^, Br^–^, Cl^–^) or a mixed halide [60, 61]. Here, perovskite is also known as organic–inorganic hybrid perovskite. However, organic components are easily affected by external factors such as light, heat, and humidity. By replacing A in ABX_3_ with inorganic cations, fully inorganic perovskite materials can be prepared with better stability [62, 63]. Xu et al. prepared a single-crystal CsPbCl_3_ UV detector using vapour-phase epitaxy, which has high sensitivity and stability [64]. Lead-based perovskites have made progress in performance, but their toxicity and stability limit further commercialization. In order to further enhance the performance of perovskites and reduce toxicity, researchers have prepared lead-free perovskites using a strategy as shown in Fig. 5 [65]. At present, researchers have also discovered that various dimensions of perovskite can be used for photodetectors, including quantum dots (0D), nanowires (1D), nanosheets (2D), and bulk crystals (3D) [66]. Perovskite oxide also has excellent performance in the field of optoelectronic detection. It can be prepared into ultra-thin layered perovskite oxide nanosheets through multi-step soft chemical exfoliation technology, with properties such as photocatalysis, magnetoresistance, and high-temperature superconductivity [60]. The optoelectronic properties of perovskite are related to its surface/interface engineering and micro-/nanostructure design. Nanowire perovskites with high aspect ratios can enhance their applications in polarization optics [67]; long chain organic ligands will limit carrier transport; excessive specific surface area will result in more material defects [68]. Moreover, perovskite polycrystalline thin films have the advantage of flexibility and are highly advantageous in the field of preparing flexible optoelectronic devices [69]. Gu et al. embedded perovskite into PDMS precursors through inkjet printing, forming perovskite arrays distributed on PDMS flexible films to prepare wearable optoelectronic devices [70].

Perovskite is popular in photodetectors for its good light absorption, tunable bandgap, low cost, good stability, low-temperature processing, and excellent optoelectronic properties. However, the ionic bonding in lead halide perovskite is affected by chemicals, and therefore, it is not possible to lithograph them using polar solvents, which limits their integration and the realization of high-resolution detectors [71].

**Fig. 6:**
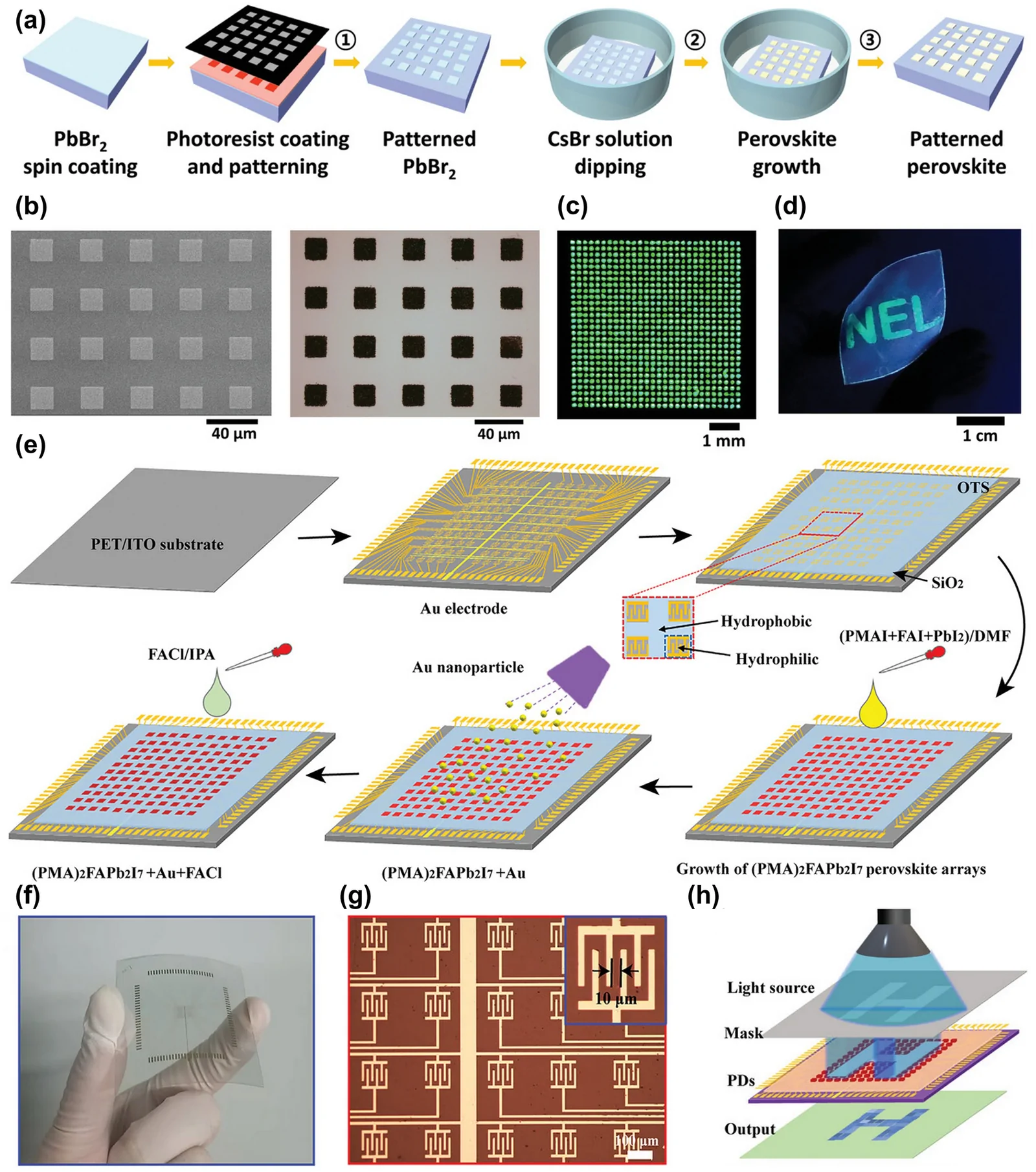
Perovskite photodetectors. **a** Process schematic of the PbBr_2_ high-resolution photodetector. **b** SEM (left: by spinning) and optical microscope (right: by − 20 °C iso-propyl alcohol) of PbBr_2_ photodetectors. **c** Photoluminescence picture of Cs_4_PbBr_6_ array. **d** Photoluminescence picture of flexible perovskite photodetector [71], Copyright 2021, Advanced Functional Materials. **e** Process schematic of the perovskite flexible photodetector. **f** Optical image of the perovskite flexible photodetector array. **g** SEM of Au electrodes. **h** Schematic of the flexible perovskite photodetector arrays to detect multipoint light distribution [76], Copyright 2023, Advanced Functional Materials

As shown in Fig. 6a–d, Kim et al. take advantage of existing lithography to synthesize Cs_x_Pb_y_Br_z_ with good light absorption performance by patterning PbBr_2_ to achieve high resolution [71]. Liang et al. also used ultrathin package-assisted lithography to prepare photodetector arrays with vertical crossbar structures with a resolution of up to 317 Pixel per inch and fully incorporated with existing lithography, solving the problem of unsuitability of perovskite polar solvent lithography [72]. Yang et al. also designed perovskite photodetectors with a resolution of up to 1 μm [73]. The destruction of perovskite by polar solvents can also be effectively prevented by applying a polymer protective layer, or with the help of self-assembled monolayer pattern templates [74, 75]. Some microcrystalline perovskites, such as MAPbBr_3_ microcrystals, have a trap state inhomogeneous distribution and spatial inhomogeneity of photocurrent, the control of which can further enhance the performance of chalcogenide photodetectors [73]. Surface interface engineering also plays a role in the enhancement of the functionality of perovskite photodetectors. Wang et al. prepared high-resolution perovskite photodetector arrays on polymer substrates with the help of silica hydrophilic and hydrophobic treatment process, which maintains good stability under bending, as shown in Fig. 6e–h [76]. The quasi-2D perovskite is constituted by incorporating large organic cations in 3-dimensional perovskites with good stability, and this composite structure allows the presence of a large number of quantum wells in the perovskite and has low binding energy and high deformation resistance, resulting in optimized charge transfer properties. However, the conductivity of quasi-2D perovskite is reduced and needs to be improved by enhancing the crystallinity and optimizing the interface [76, 77].

Perovskites have tunable energy bandgaps and thus offer unparalleled advantages in the preparation of full-colour light-detection imaging without colour filters. Constructing continuous structures with graded bandgaps and pixelated structures with discrete bandgaps are commonly used to achieve full-colour imaging, with the former being limited in terms of batch size and the latter requiring fine microcell design [78, 79]. Zhou et al. prepared self-powered polycrystalline perovskite photodetectors, which suppressed the dark count rate mainly through the reduction of shallow traps' particle size enhancement effect and surface passivation and thus dramatically enhance the response to weak light, with better weak light responsivity than silicon photomultiplier tubes [80]. The preparation of flexible single-crystal perovskite photodetector devices has been realized [14]. Both cost and compatibility need to be considered in practical applications to facilitate the commercialization of perovskite photodetectors. Mahato et al. developed a MAPbBr_3_ crystallisation technique through surface/interface engineering that is capable of generating high-quality single crystals with improved photogenerated carrier separation and transfer [81].

#### Transition-metal Dichalcogenides

Transition-metal dichalcogenides (TMDCs) have tunable band gaps, high mobility, good stability, easy accessibility, and Schottky junction photodetectors based on high detection rates and fast response [82]. Molybdenum disulphide has a tunable indirect bandgap of about 1.3–1.8 eV and transforms from an indirect bandgap to a direct bandgap in a single molecular layer [18]. For molybdenum disulphide, CVD and mechanically stripped prepared photodetectors have better response rates than other methods such as magnetron sputtering, liquid exfoliation, solution synthesis, and so on [18]. Molybdenum disulphide photodetectors are influenced by both photochemical and photoconductive effects. Its photogating effect is slower, influenced by the trapping charge at the interface and modulated by the gate voltage, while the photoconductive effect of it is very rapid and is caused by structural defects in the material [18]. Monolayer MoSe_2_ is also a typical material for photo-detecting TMDCs, with a direct bandgap of 1.5 ev [83]. WS_2_ is also a photo-detecting material that is photon-energy and the surrounding gaseous environment dependent; gas molecules such as ammonia are able to increase the material's charge transfer [84, 85]. 2DWSe_2_ also has good photoconversion capabilities. MoTe_2_ is a newer 2D photovoltaic material. For TMDC materials, the reduction of charge impurities and defects is important for performance enhancement.

##### Re-dichalcogenide

Re-dichalcogenides such as ReS_2_ and ReSe_2_ differ from TMDCs with higher lattice symmetry [18]. The physical properties of these materials are anisotropic and affect the material properties in photovoltaic conversion through thickness [86]. The surrounding gas has a great influence on the performance of this photodetector, which affects the doped carrier migration through charge transfer and needs to be improved by encapsulation. However, the dark current of ReSe2 does not return to its original level after one cycle, which affects its practical application, a dilemma that can be ameliorated by applying a short pulse reset device at the gate [18, 87]. The anisotropic crystal structure of Re-dichalcogenides makes them highly advantageous for detecting polarized light.

##### Noble Metal Dichalcogenides

Noble TMDCs are a category of TMDC materials composed mainly of Group-10 noble metal elements. The d electrons of this category of materials completely occupy the d orbitals, leading to strong interlayer interactions and p-orbital hybridization [88, 89]. NTMDCs have a small bandgap but a wide tunable range and gradually overshoot the metallic state with the increase in the number of layers. They also have high mobility and environmental stability [18]. Among them, PdS_2_ and PdSe_2_ have folded pentagonal anisotropic structures, which are very promising for polarized light detection. Noble metal dichalcogenides have high mobility at room temperature with a controllable number of layers, which is highly advantageous in the near-infrared wavelength detection region, and the performance of the detectors may be enhanced by plasma treatment, etc. [90].

#### Other Photoelectric Materials

##### Group III-Nitrides

Compared to other semiconductor materials, nitride semiconductor materials are more environmentally friendly, with non-toxic preparation processes [18]. Indium nitride (InN), aluminium nitride (AlN), and gallium nitride are semiconductor materials with tunable bandgap for industrial applications, which are valuable in photodetectors. However, epitaxial growth and substrate matching of Group III-Nitrides are still a challenge, also costs, etc., still need to be further reduced [18].

##### Metal Oxide

Metal oxide include titanium dioxide, tin oxide, zinc oxide, tungsten trioxide, iron trioxide, and others. Tin oxide is an N-type semiconductor with a strong oxidizing ability and has a wide range of applications in the fields of optoelectronics, gas sensing, and catalysis [91]. Tin oxide has a band gap of 3.7 eV and limited UV absorption, which can be improved by surface interface modification and design of components [91].

##### Metallic Materials

Metals perform the photoelectric conversion function in photodetectors mainly through Schottky junctions. Absorption of photons by metals results in the generation of hot carriers, which are injected into the semiconductor and form a current at the interface of the junction through the junction barrier [92]. By adjusting the size and length-to-diameter ratio of metal nanorods, nanoparticles, heptamer arrays, gratings, etc., the absorption bands can be tuned to improve the response of the device [92, 93].

##### Lanthanide Materials

Lanthanide crystals have a high number of discrete energy levels and can trap incident vortex-wave light and emit visible light, which are known as upconverting nanocrystals. In order to enhance the light absorption and conversion of lanthanides, it is crucial to overcome the low extinction coefficient and small size-induced surface burst effect [92]. It can also be improved by designing doping, customizing the core-shell structure, and introducing photonic and plasmonic crystals [92]. In addition, 2D materials, especially 2D dielectrics, piezoelectrics, pyroelectrics and ferroelectrics, and their interactions and coupling synergies with optoelectronics are of great importance. Xing et al. have proposed a variety of solutions to optimise light detection by comprehensively exploring the physical coupling mechanisms of these materials [94].

In photodetectors, the material energy gap determines the band of light that can be detected. Currently, various semiconductor materials cover the range from the ultraviolet to the far-infrared, and corresponding photosensitive materials can be found for each band [17]. Optimized semiconductor devices can be discovered by continuously exploring new materials. Understanding the characteristics and advantages of various materials can also help us to choose more appropriate and ideal materials when preparing optoelectronic devices, which can inspire the discovery of new optoelectronic materials.

### Manufacturing Process

The preparation of optoelectronic materials is a complex process involving precise tracking of material evolution, accurate manipulation of complex units, and a batch process that involves many aspects such as cost, energy saving, environmental protection, and product quality, which is a key component of high-resolution optoelectronic devices. High-resolution optoelectronic devices imply smaller feature sizes, i.e. nodes, which are the smallest individual photovoltaic units that can be produced by the manufacturing process [95]. The higher the resolution, the more optoelectronic units will be available on the same size chip, reflecting the optical signal more accurately and utilizing the light energy efficiently. Intel can only manufacture integrated circuits with a minimum node of 14 nm [95], the current state-of-the-art process is a 2-nm feature size chip, and Samsung plans to put it into production in 2025 [95].

The focus of optoelectronic chip preparation is to combine the circuitry, the optoelectronic material and the substrate in turn, to form the predefined node distributions. Typically, wafers (150–450 mm in diameter) or other substrates are first oxidized and heated in an oxidizing atmosphere furnace, after which thin-film photovoltaic materials are deposited on the wafers through various coating preparation processes; the coatings are then made in a predetermined pattern through photolithography. This process involves applying a photoresist to the surface of the material and then applying a mask with a specific pattern, so the coating is patterned by photolithography. In production, different photolithography procedures may be carried out dozens of times to form the functional structure. Finally, the photoresist is removed and a metal or other material is deposited to connect the functional units [95].

#### Atomic Layer Deposition

**Fig. 7:**
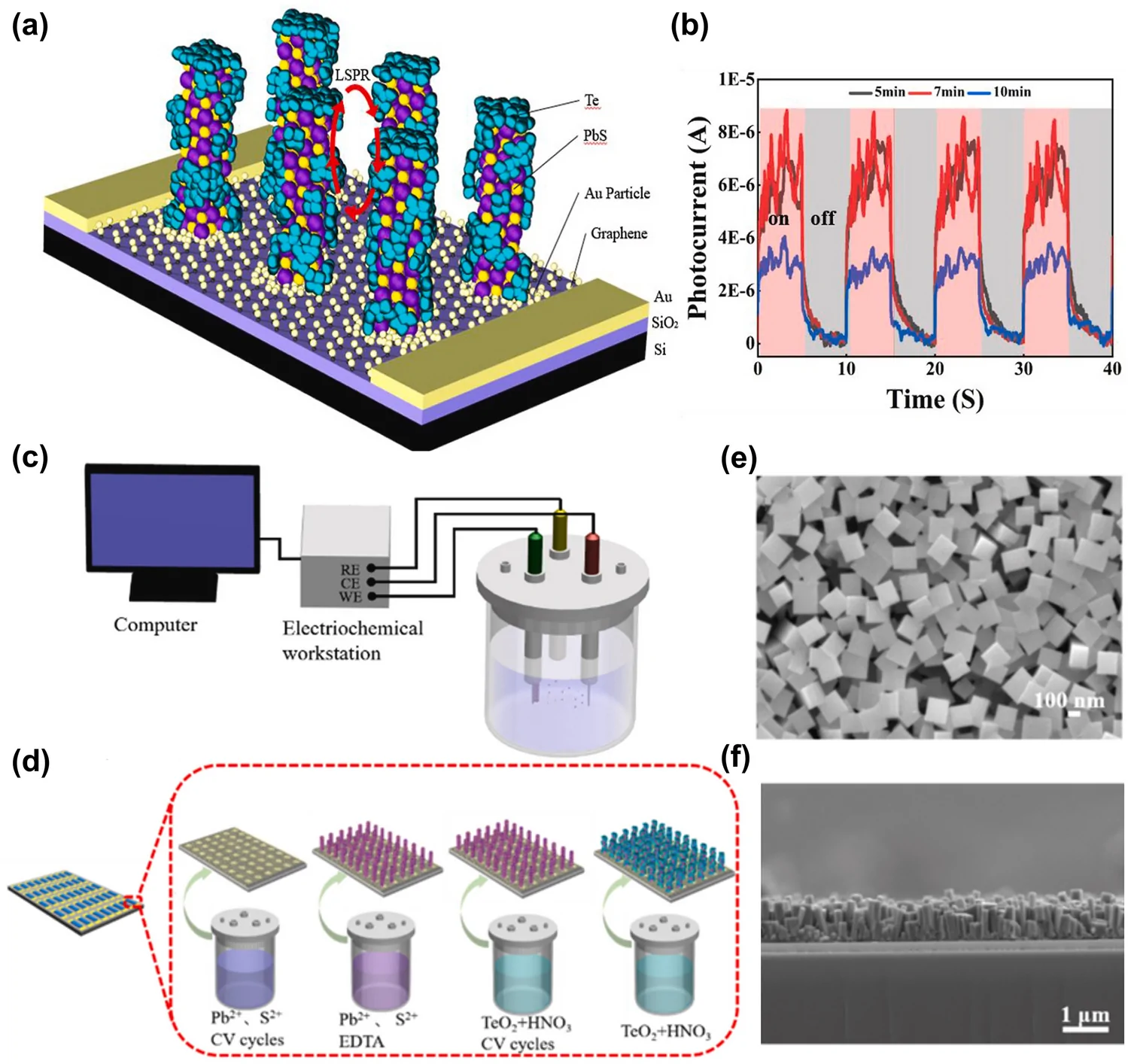
Photodetectors prepared electrochemical atomic layer epitaxy [96], Copyright 2023, Physica E: Low-dimensional Systems and Nanostructures. **a** Schematic of Te/PbS composite photodetector. **b** Photocurrent response of Te/PbS photodetector in different deposition time. **c** Electrochemical workstation. **d** Electrochemical deposition. **e** PbS nanorods top view. **f** PbS nanorods cross-section

Atomic layer deposition (ALD) is a thin-film preparation technique based on layer-by-layer atomic-level growth, which allows for the deposition of ultrathin films of uniform, controllable thickness and adjustable composition. The remaining unreacted precursors have to be blown away with an inert carrier gas in between the ALD layer and layer, to avoid gas-phase reactions. Zhang et al. prepared PbS nanorods vertically on graphene layers via electrochemical ALD, demonstrating superior optoelectronic properties over layered PbS (Fig. 7) [96]. Jiao et al. also prepared alumina interfacial layers by ALD to avoid the reaction at the interface of the heterogeneous material, thus improving the optoelectronic properties [97].

#### Physical Vapour Deposition

**Fig. 8:**
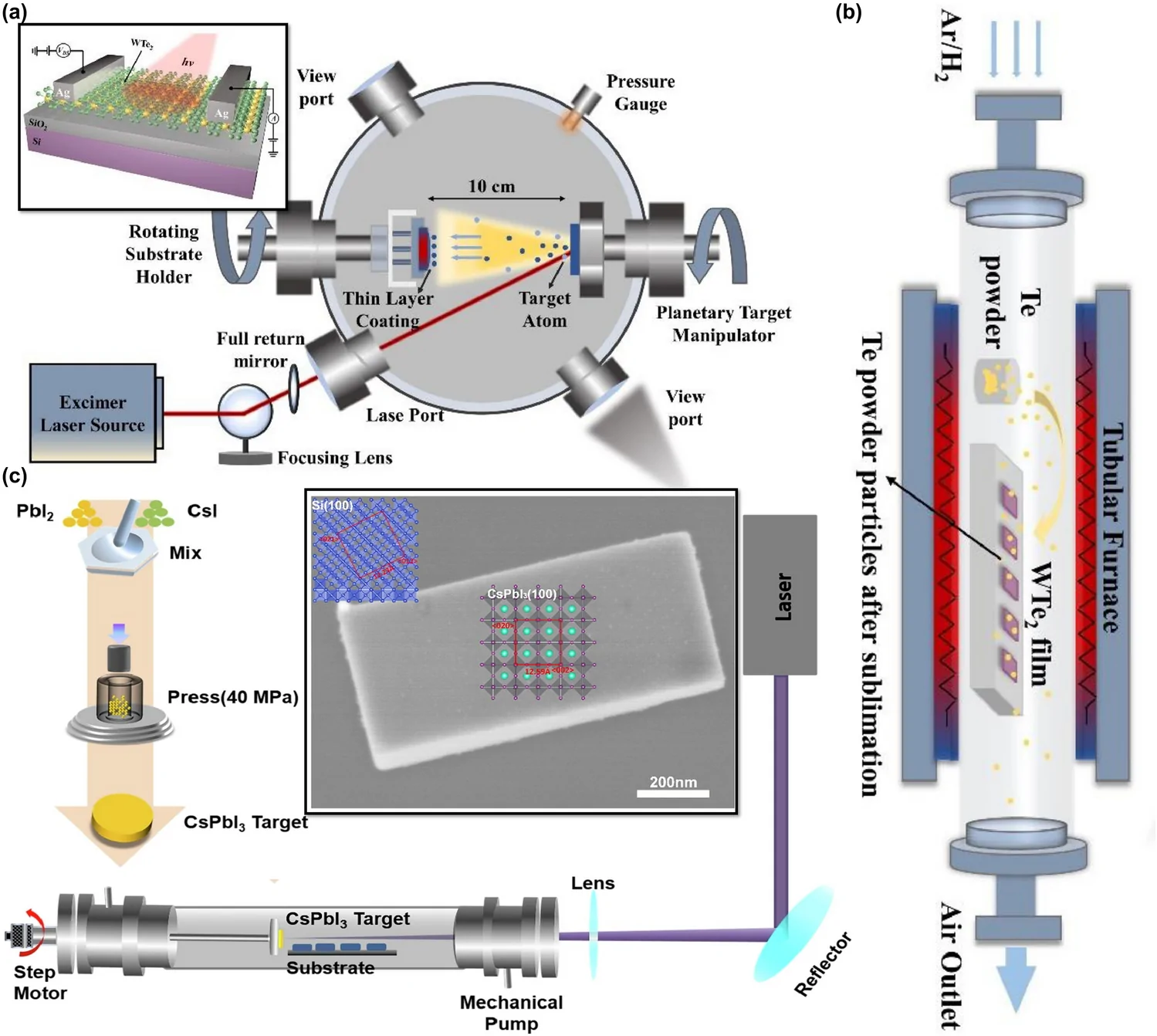
Photodetectors prepared PLD. **a** Schematic of WTe_2_ photodetectors prepared by PLD (Insert image is photodetector structure). **b** Schematic of WTe_2_ post-annealing treatment [101], Copyright 2024, Surfaces and Interfaces. **c** Schematic of high pressure PLD (Insert image is a-CsPbI_3_ nano-brick made by pressure PLD) [103], Copyright 2022, Journal of Colloid and Interface Science

Physical vapour deposition (PVD) refers to the technique of depositing thin films on the surface of a substrate by physically vaporizing a material source into gaseous atoms or molecules, or partially ionizing them into ions, and depositing them on the surface of the substrate by means of a low-pressure gas (or plasma) under vacuum conditions. Physical vapour deposition acts primarily on the surface and includes magnetron sputtering, molecular beam epitaxy (MBE), pulsed laser deposition, and so on. Magnetron sputtering is a commonly used PVD method. Solovan et al. coated N-type molybdenum oxide on silicon substrates with nanostructures to prepare optoelectronic heterojunctions by this method [98]. MBE has a lower synthesis temperature, is greener, and has high product quality compared to other vapour deposition [18]. It grows semiconductor materials epitaxially and layer-by-layer by precisely controlling the thermal evaporation of atomic substances to form lattice-matched 2D layers [99]. 1D zinc oxide, etc., can also be obtained by MBE [100]. Moreover, it is controllable, scalable and highly advantageous in the preparation of highly pure materials. With the development of techniques such as plasma-assisted molecular beam epitaxy (PAMBE), the synthesis time has been further reduced. Pulsed laser deposition (PLD) is a means of obtaining thin films by using a laser to bombard an object and then precipitate the bombarded substances on different substrates. The structure of the device is shown in Fig. 8a, b [101]. Xiao et al. prepared a large-area (10 × 10 mm^2^) WTe_2_ film by PLD, which has low impurities and defects, uniformity, and high quality, and is conducive to photovoltaic properties improvement. WTe_2_ was further improved after annealing at 400 degrees Celsius [101]. Kang et al. prepared VO_2_/glass composite membranes with a titanium dioxide buffer layer by PLD, and the presence of titanium dioxide improved the original photovoltaic properties [102]. Zhou et al. prepared stabilized and controllable nano-bricks on a silicon substrate by high-pressure PLD and demonstrated great potential for applications in the field of photovoltaics, as shown in Fig. 8c [103].

#### Chemical Vapour Deposition

**Fig. 9:**
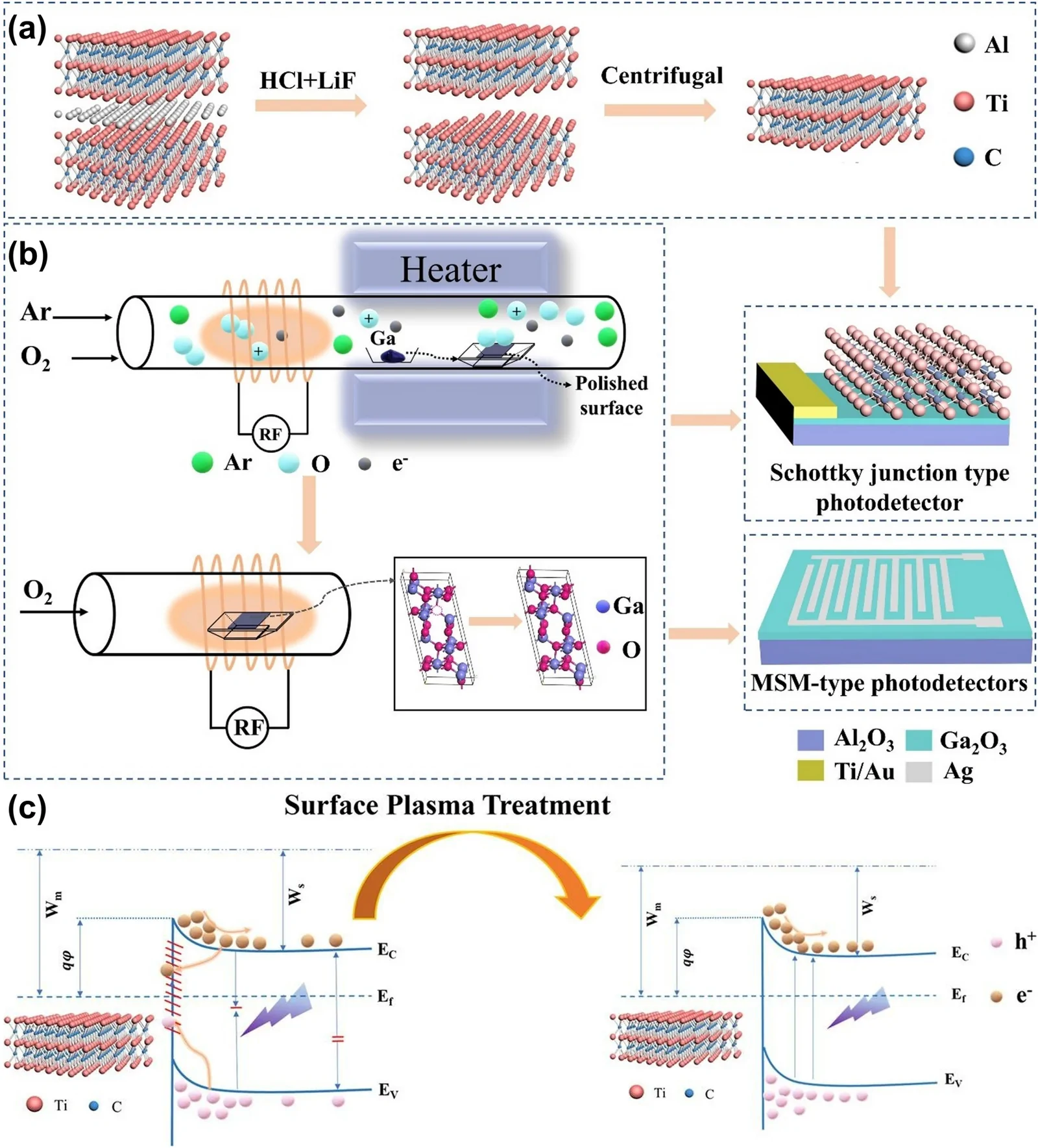
Photodetectors prepared plasma-enhanced CVD [104], Copyright 2022, Applied Surface Science. **a**, **b** Oxygen plasma treatment of β-Ga_2_O_3_ to make a Ti_3_C_2_/β-Ga_2_O_3_ self-powered photodetector. **c** Energy band changes before (left) and after (right) oxygen plasma treatment

Chemical vapour deposition (CVD) is a widely used technique for the synthesis of photovoltaic materials, which has a wide range of applications due to its high controllability and the ability to prepare large-size 2D materials. Mu et al. prepared tetragonal nano-Schottky heterojunctions of tin oxide by CVD and modified gold nanoparticles on their surfaces by further hydrothermal method to enhance the photothermal performance through localized plasma oscillations [91]. Deng et al. used plasma-enhanced CVD to prepare gallium oxide thin films to construct Schottky junction-based photodetectors, and the photocurrent was enhanced more than five times after plasma treatment, as shown in Fig. 9 [104]. Jiang et al. also prepared nanowire/nanosheet heterostructures for photodetection by a combination of magnetron sputtering and CVD [105]. Li et al. used CVD for finely tuned growth of transition metal dihalides with different compositions to form lateral heterojunctions with sharp interfaces [106]. In order to enhance the commercial production compatibility of high-quality perovskite thin films, Reo et al. prepared perovskite thin-film transistors through thermal evaporation method, significantly improving the density and uniformity of the material and enhancing device performance [107].

Chemical vapour transport (CVT) is also a traditional method of crystal growth by setting up a quartz furnace with a strict ratio of platinum and selenium powders and allowing the gas precursor to undergo a vapour reaction and be deposited elsewhere under high temperatures and vacuum conditions, with good controllability [90].

#### Vapor-phase Epitaxy

Vapour-phase epitaxy, a form of CVD, is a common method for preparing two-dimensional, thin-layer, and high-quality semiconductor materials. As early as the 1960s, Maruska et al. used hydride vapour-phase epitaxy to prepare single-crystal gallium nitride layers [108]. In addition, metal–organic chemical vapour deposition (MOCVD) and metal–organic vapour-phase epitaxy (MOVPE) have also been used for the batch preparation of semiconductor layers. However, the higher reaction temperatures of these preparation processes lead to stress strains during cooling, resulting in quality degradation [18].

#### Spray Pyrolysis

**Fig. 10:**
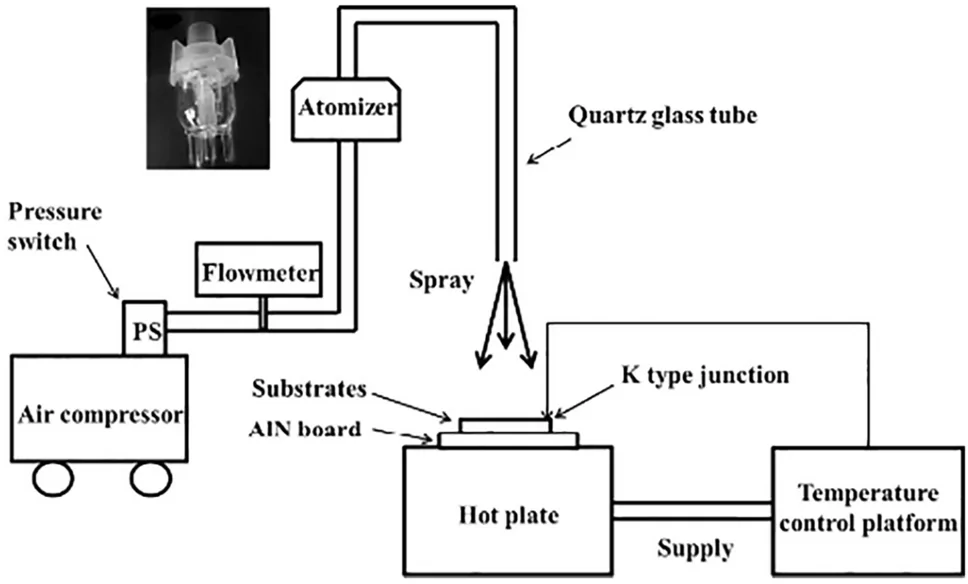
Schematic of spray pyrolysis device [109], Copyright 2019, Surface & Coatings Technology

Spray pyrolysis refers to spraying a solution in mist form into a high-temperature atmosphere, causing evaporation of the solvent and thermal decomposition of the solute, followed by supersaturation and precipitation of the solid phase, thus obtaining nanopowders. Its equipment design is shown in Fig. 10 [109]; Yu et al. improved the traditional spray pyrolysis method for the preparation of fluorine-doped tin oxide with low efficiency and high exhaust emission by adding hydrogen peroxide into the precursor gas. The addition of hydrogen peroxide not only improved the preparation process, but also narrowed the band gap and thus regulated the photoelectric properties [109]. α-Fe_2_O_3_ was also coated on the substrate and used for UV photodetection by Kaawash et al. by scalable spray pyrolysis [110].

#### Electrohydrodynamic (EHD) Printing

**Fig. 11:**
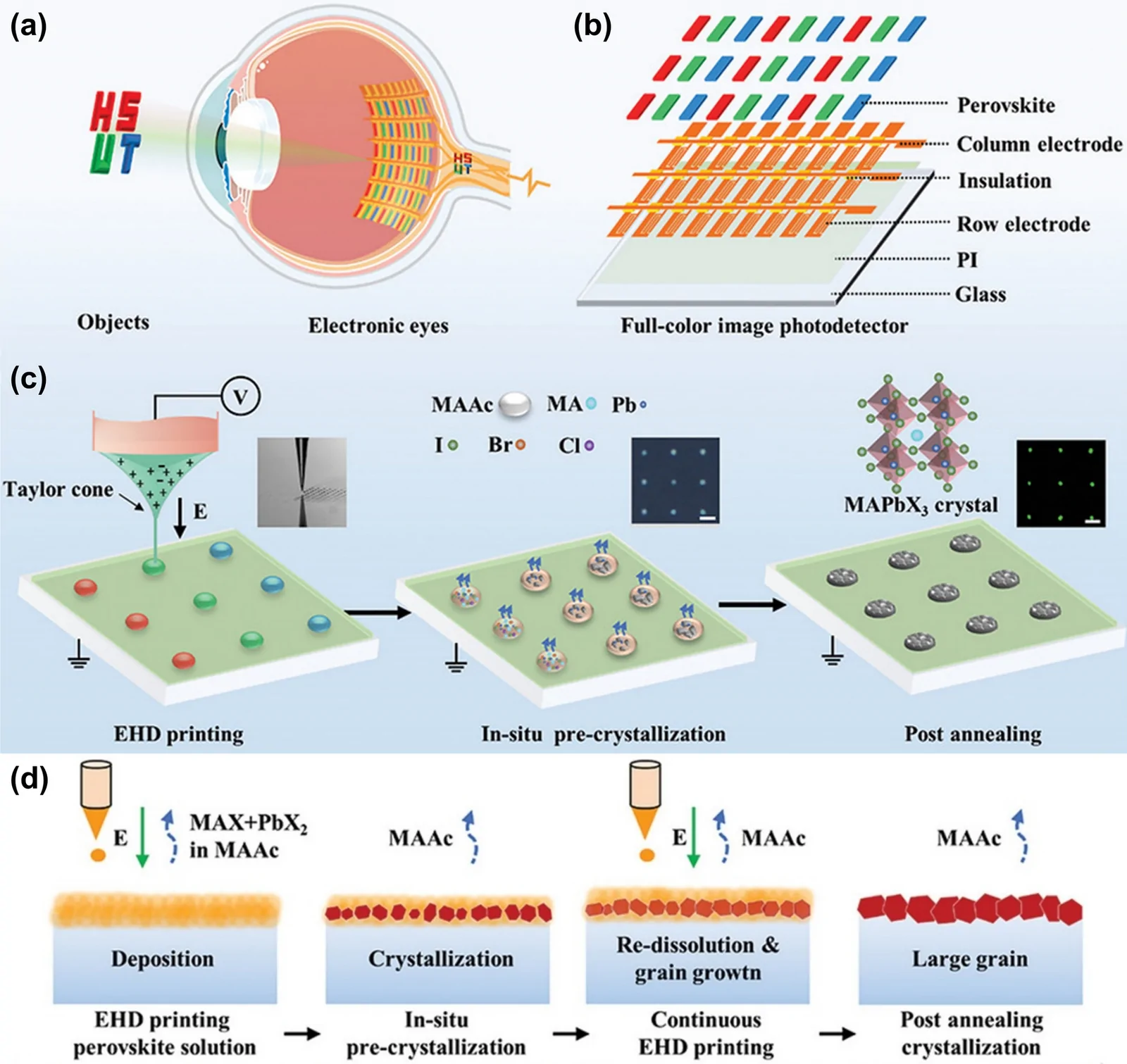
High-Resolution, flexible, and perovskite photodetector via EHD printing [113], Copyright 2021, Advanced Functional Materials. **a** The human like eye function of high-resolution light detection arrays. **b** Schematic of full-colour perovskite photodetector. **c** Schematic of the EHD printing process for photodetector array. **d** Mechanism of the EHD printing process

High-resolution optoelectronic detection devices made of flexible materials are essential for biosensing and soft robotics because of their excellent information recognition, image acquisition, and ability to cooperate with braking systems. Flexible optoelectronic devices, especially their large-area manufacturing, are a formidable challenge, a flexible preparation process is the basis for the development of flexible optoelectronic devices. The casting method is difficult to achieve extremely thin thicknesses, limiting flexible applications [111], and the self-coating method is difficult to regulate the distribution of detection units in different frequency bands [112], so accurate and efficient flexible photodetector preparation processes are important. Electrohydrodynamic printing with high resolution enables the direct printing of photoresponsive materials onto the target substrate through liquid mixtures and the control of crystal growth through appropriate solvents to reduce defects and improve quality [113]. Wang et al. prepared perovskite photodetector arrays with a resolution of 1 μm, which is the smallest printable feature size for perovskite applications, with a high responsivity of up to 14.97 A W^-1^ and a ones detectivity (D*) of 1.41 × 10^12^ J. Also, the detector is capable of full-colour light detection without the need for a colour filter by integrating different chalcogenides with a variety of spectral responses, as shown in Fig. 11 [113].

#### Hydrothermal Method and Solvothermal Reaction

**Fig. 12:**
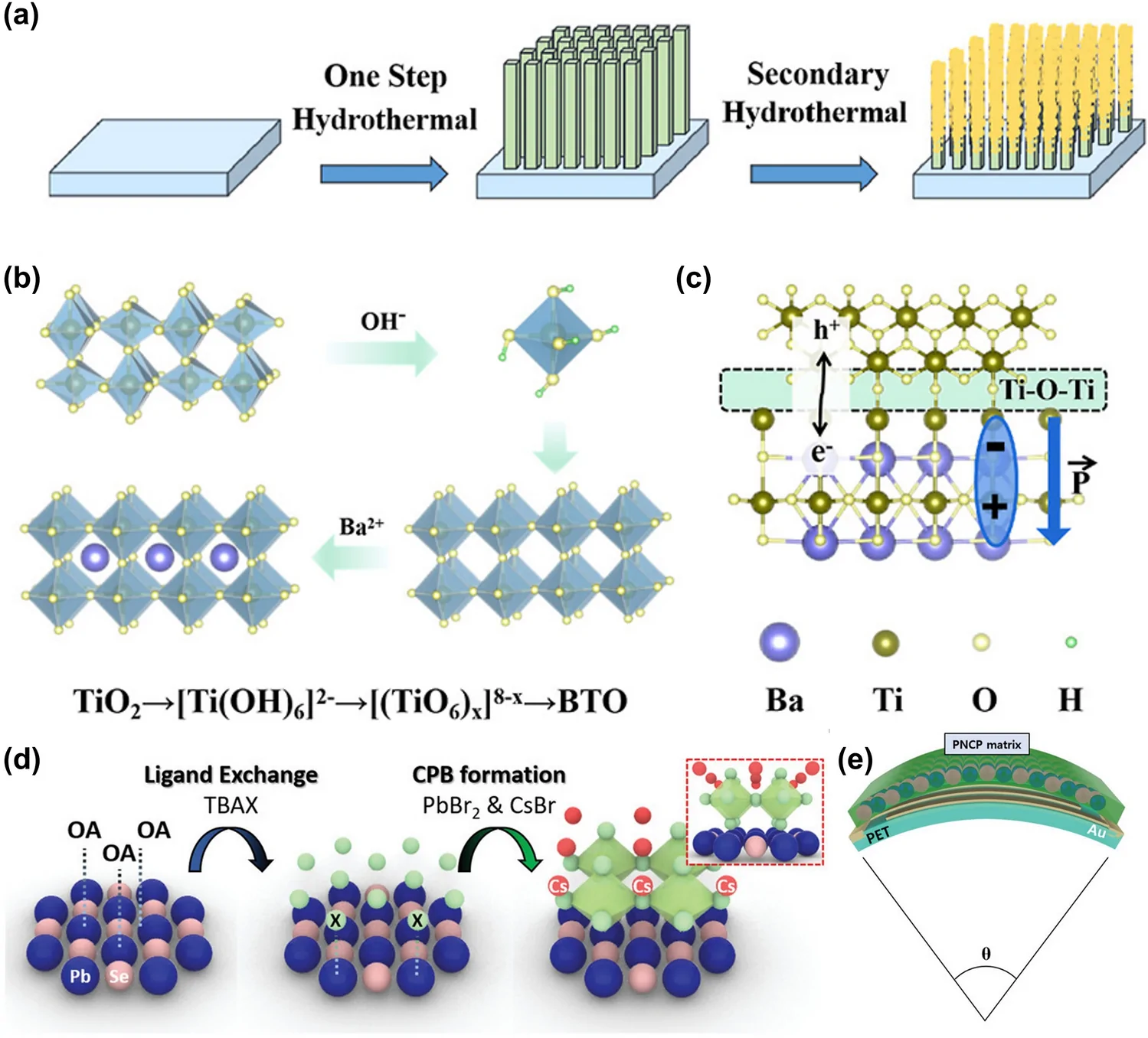
Preparation of photodetectors by hydrothermal method and halide surface chemistry, **a** fabrication process of TiO_2_-BaTiO_3_ nanorods by hydrothermal method. **b** Process of BaTiO_3_ attached to TiO_2_ nanorods. **c** Mechanism of interface engineering for the TiO_2_-BaTiO_3_ photodetector [115], Copyright 2023, ACS Applied Materials & Interfaces. **d** Schematic of the TBAX-treated PNCP prepared by halide surface chemistry (inset: front view; TBAX = tetrabutylammonium halide (X = Cl, Br, or I); PNCP = PbSe nanocrystal s in a Cs_4_PbBr_6_ perovskite). **e** The TBAC-treated PNCP photoconductors on flexible substrate [118], Copyright 2023, Advanced Optical Materials

The hydrothermal method is a process in which a solid is dissolved in water and then precipitated in a sealed container, which is often used to prepare nanocrystals with special structures. Liu et al. prepared SnS_2_ nanoflake/CdS nanorod heterojunction composite photoanodes by a two-step hydrothermal method [114]. The solvothermal reaction is based on the hydrothermal reaction by making an organic solvent instead of water [100]. Wang et al. also used two-step hydrothermal method to enhance the photoelectronic performance of TiO_2_–by-BaTiO_3_ surface modification (Fig. 12a–c) [115].

Electrochemical deposition can significantly enhance the growth rate [100].

The sol–gel method is based on the same principle as the hydrothermal method and produces a composite of the target substance with a salt, which needs to be calcined to remove the salt so has fewer applications [100]. Based on this, there is also the sol-combustion method, Yang et al. prepared fluorine-doped ZnO membranes by low-temperature sol-combustion method, and by regulating the precursor and doping concentration, photovoltaic membranes with good crystallinity and 80% light transmittance can be obtained [116].

Assisted filtration relies on the principle that the vaporization temperatures of oil and water are different under the same pressure (vacuum). Si et al. prepared molybdenum disulphide and WSe_2_ nanosheets by liquid-phase stripping followed by vacuum-assisted filtration to form heterogeneous photovoltaic electrodes with optimized photovoltaic properties [117].

#### Spin Coating

Perovskites have great potential for optoelectronic devices due to their ease of preparation and moulding by solution methods. Coating perovskites on a substrate by spin-coating on the basis of a precursor is a low-cost, inexpensive and efficient method [3]. Traditional chemical deposition can also be used for surface modification and the preparation of photodetectors, as shown in Fig. 12d, e [118].

#### Self-assembly

**Fig. 13:**
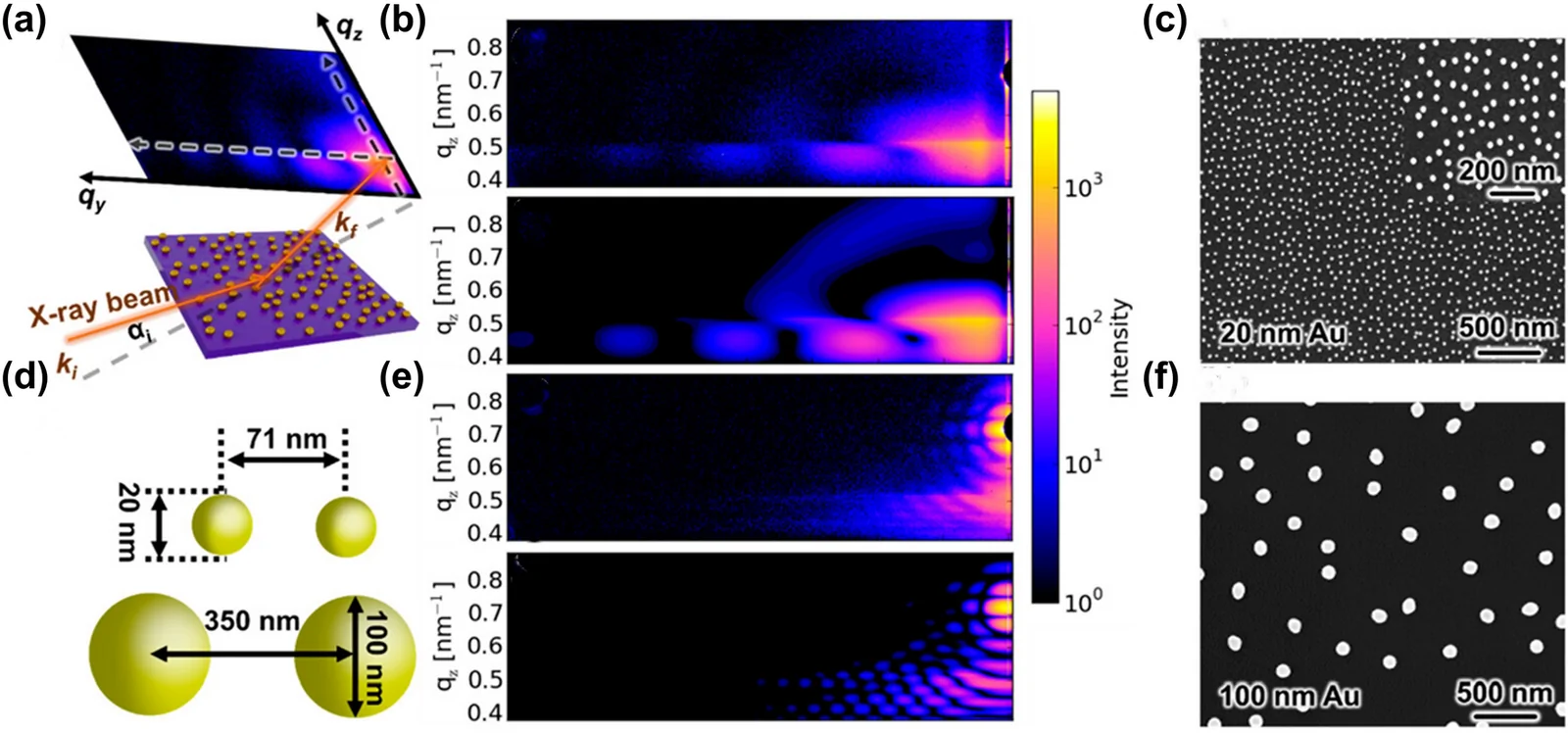
Plasmonic interface structure photodetector by self-assembly [124], Copyright 2023, ACS Nano. **a** Grazing-incidence small-angle X-ray scattering setup for detecting nanostructures and interfaces by self-assembly. **b** 20 nm Au nanospheres monolayers (above) and their simulation results (below) via BornAgain. **c** 20 nm Au nanospheres monolayers. **d** Grazing-incidence small-angle X-ray scattering simulation parameters of (upper) 20 nm and (bottom) 100 nm. **e** 100 nm Au nanospheres monolayers (above) and their simulation results (below) via BornAgain. **f** 100 nm Au nanospheres monolayers

Self-assembly is a technique for assembling disordered nanoparticles into ordered and arranged films, which mainly relies on molecular interaction forces, and the introduction of an externally induced source can enhance the self-assembly effect [119]. Liu et al. prepared CuTCPP(Cu)/CuTCPP(Fe) heterojunctions by electrostatic self-assembly, whose π–π coupling inhibits carrier recombination and thus improves the optoelectronic properties [120]. Langmuir–Blodgett technique is usually used to construct coatings on liquid–gas interfaces, through which spontaneous assembly of micro-/nanoparticles can be realized, capable of forming coatings on liquid–gas interfaces, which is highly advantageous in the preparation of monolayers or controllable multilayers [119]. Li et al. achieved dye molecule/polypeptide composite membranes with good photocurrent through the Langmuir–Blodgett self-assembly technique by means of electrostatic, hydrogen bonding, π–π stacking, and other synergistic effects, which showed potential in alkaline gas sensing and light detection [121]. Dye molecule/carbon sphere composite membranes prepared by this technique also showed good photocurrent density and carrier transport [122]. Sui et al. prepared graded photovoltaic materials by ionic self-assembly, which were able to respond to redox stimuli, pH changes, etc., and showed graded features [123]. Guan et al. also prepared plasmonic interface structure photodetector by self-assembly, as shown in Fig. 13 [123].

#### Photolithography

**Fig. 14:**
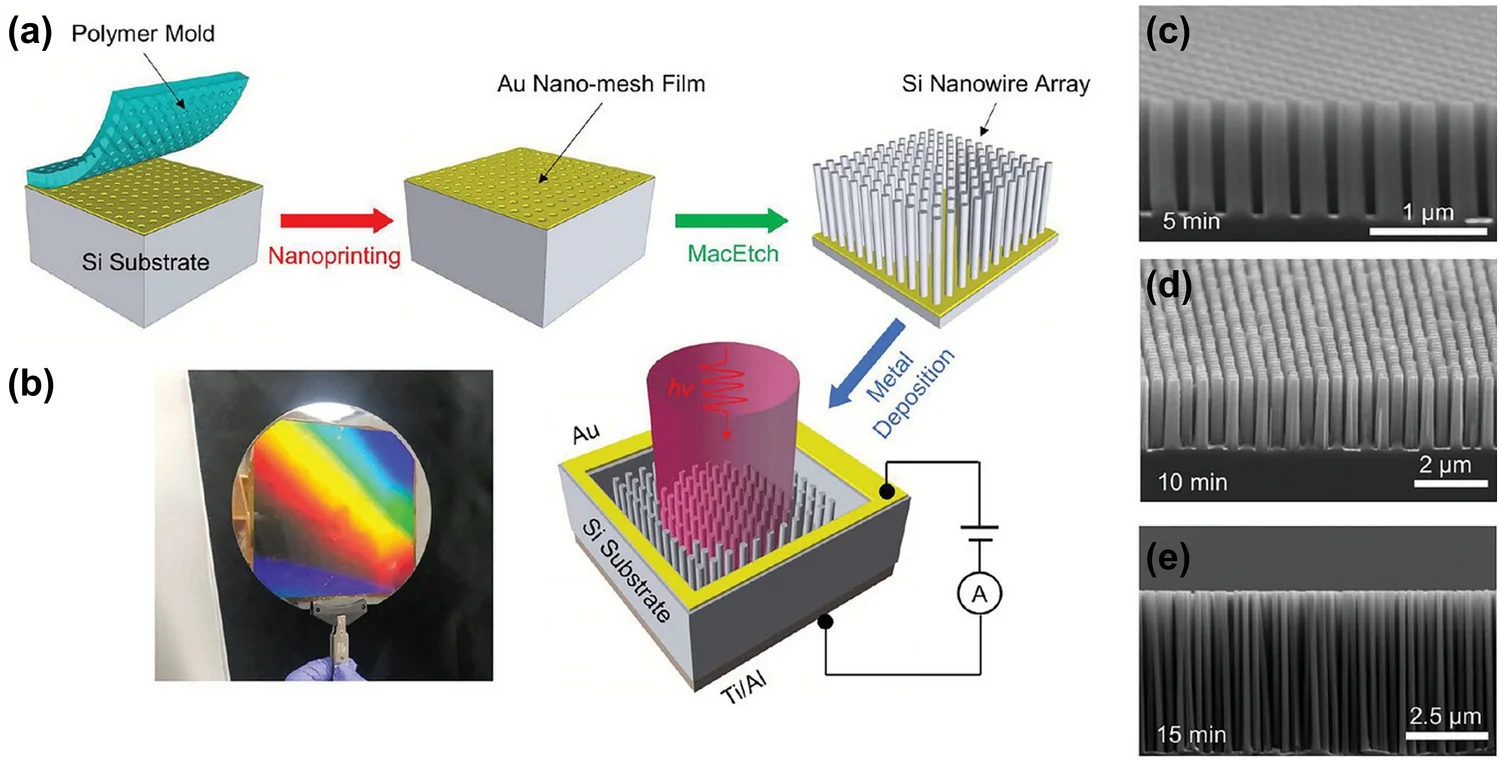
Manufacturing of photodetectors by nanotransfer printing and MacEtch techniques [125], Copyright 2023, Advanced Materials Technologies. **a** The fabrication process of the Si nanowire array photodetector by nanotransfer printing. **b** Picture of the 6 in. Si nanowire array. Cross-sectional SEM of Si nanowire arrays after the MacEtch for **c** 5 min, **d** 10 min, and **e** 15 min

Photolithography is generally only able to etch nodes of the same wavelength size as itself, and as node sizes decrease, shorter wavelengths of light are required, so photolithography is progressively more limited, requiring techniques such as electron beam (e-beam) lithography and EUV lithography to achieve smaller feature sizes [95]. Micro-/nano-printing based on patterned polymer films has also been used for the preparation of photodetectors (Fig. 14). Therefore, innovation in lithography is challenging and critical for high-resolution optoelectronic devices.

The properties of 2D materials are greatly influenced by the preparation process, and it is important to choose the appropriate manufacturing process according to different materials. For example, in chemical vapour deposition, molybdenum disulphide with a large specific surface area is highly susceptible to adsorption of other impurities in the gas, which leads to scattering and reduces the performance of the photodetector [126, 127]. Researchers are also exploring new preparation processes to improve the quality, micro-nano control, and size of semiconductor materials. For example, epitaxial lateral overgrowth can be used to optimise the semiconductor structure with the help of a buffer layer [18]. Exploring better substrate materials or designing microstructures of the substrate by quantum confinement is important for improving the quality of products [18].

Different materials have their own more suitable manufacturing process, the choice of the appropriate process can maximise the advantages of each material, and more precise preparation technology is also conducive to the realization of smaller size nano-optoelectronic components. However, the development of preparation technology is usually limited by the size, the development of micro-/nanofabrication equipment urgently needs the innovation of related technologies, such as photolithography nodes are affected by the wavelength of light, high-frequency lithography can promote the development of small-scale optoelectronic devices. In addition, the post-processing processes, annealing, UV radiation, etc., are also very critical to improve the performance.

## Surface/Interface Engineering

Optoelectronic semiconductor devices are often faced with limited absorption bands, low energy conversion, fast carrier annihilation, and poor interfacial charge transfer due to the limitations of material densities, grain sizes, preparation processes, and the characteristics of the materials [128, 129]. In order to improve these deficiencies, it is of great significance to modify the surface/interface of the optoelectronic device or design the surface/interface micro-/nanostructures through the surface/interface engineering, to improve the photoelectric conversion efficiency and broaden the response band, which is important for the improvement of the performance of high-resolution optoelectronic devices.

### Surface/Interface Modification

**Fig. 15:**
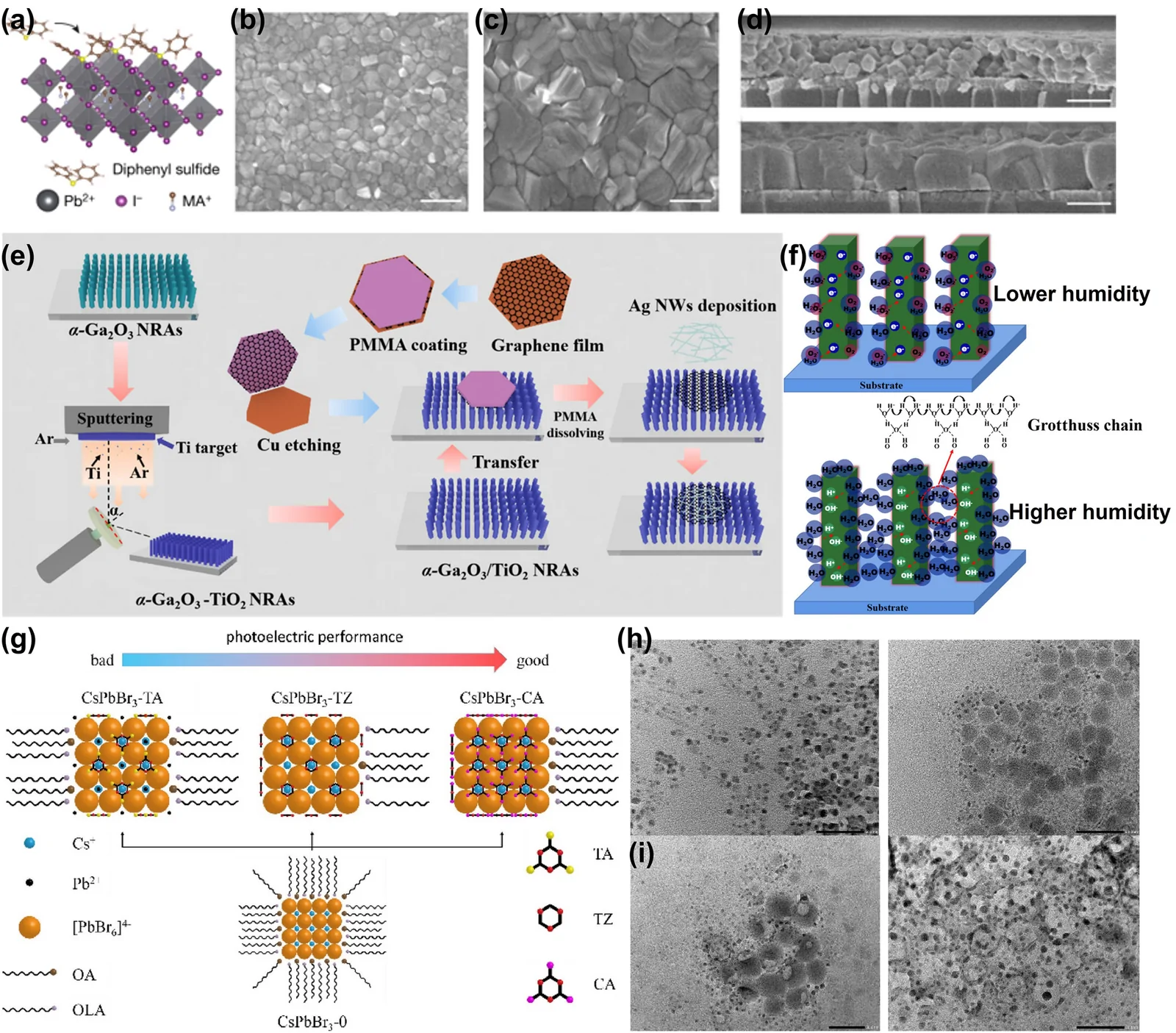
Optoelectronic performance optimization through surface/interface modification or passivation. **a–d** Reducing deep traps by morphology optimisation and surface diphenyl sulphide passivation modification. **a** Surface defect passivation by diphenyl sulphide. **b** Top-view SEM of one-step MAPbI_3_ film. **c** Top-view SEM of two-step MAPbI_3_ film. **d** Cross-sectional SEM of one-step MAPbI_3_ film [80], Copyright 2023, Nature. Optoelectronic performance optimization by passivation: **e** Fabrication of the α-Ga_2_O_3_ − TiO_2_ core − shell nanorod array solar-blind photodetector with a graphene − Ag NW hybrid film as the top electrode; **f** Mechanism of humidity on the photoelectric performance [134], Copyright 2022, ACS Applied Electronic Materials. Optoelectronic performance optimization by s-triazine and its derivatives surface modification: **g** Surface modification for CsPbBr_3_ (TA = trithiocyanuric acid; TZ = s-triazine; CA = cyanuric acid); **h** TEM of CsPbBr_3_-0 (left) and CsPbBr_3_-TA (right). **i** TEM of CsPbBr_3_-TZ (left) and CsPbBr_3_-CA (right) [141], Copyright 2022, Chinese Chemical Letters

Functional groups and surface defect modifications can change the physical and chemical properties of the materials, and for small sizes especially 2D photovoltaics, the impact of such modifications on the photovoltaic properties of the material can be dramatic [130]. The photoresponse of metal dichalcogenides such as molybdenum disulphide and ReS_2_ can be improved by surface modifications such as oxidation and doping [131]. For high specific surface area molybdenum disulphide, encapsulation can also reduce its undesirable adsorption, thereby enhancing mobility and lowering contact resistance for improved performance [132]. By controlling the morphology and defect passivation, the metal halide perovskite deep traps can be effectively reduced, thus avoiding the large dark count rate caused by the presence of deep traps. Zhou et al. reduced the deep traps by morphology optimisation and surface diphenyl sulphide passivation modification, so shallow traps play a dominant role in enabling the photodetectors to achieve a pulse detection probability of more than 99.8% and an internal quantum efficiency is also up to more than 90%, with better resolution than commercial silicon photomultiplier tubes at room temperature, as shown in Fig. 15a–d [80]. Moreover, the addition of PbI_2_ to the precursor, and the evaporation of CH_3_NH_3_I caused by over-annealing can also passivate the perovskite [133]. Due to the defects in α-Ga_2_O_3_ nanorod array optoelectronic devices, the optoelectronic performance can be enhanced by using wide bandgap semiconductor materials such as titanium dioxide and alumina, as passivation layers. In addition, the type ii staggered-band arrangement between them also can effectively separate the photogenerated carriers and thus promote the photovoltaic conversion [134]. In this study, humidity also affects the photovoltaic performance of the device, and in a high humidity environment, strong chemical bonds are formed between water molecules and trivalent titanium ions on the surface of the nanorod arrays, generating a large number of plasmons and thus enhancing the electrical conductivity, as shown in Fig. 15e, f. Taha et al. systematically illustrated the positive effects of the synergistic effect of plasma nanostructures, especially metallic wire structures on light absorption and charge transfer, and explored the tailoring of such structures [135]. Relying on the self-passivation behaviour of TaSe, Wang et al. prepared highly stable photodetectors capable of resisting oxidation [136]. Punia et al. also dramatically improved light trapping through interface engineering by passivating the silicon surface and designing microtextures [137].

With the development of flexible optoelectronic devices, the mismatch between organic and inorganic interfaces in optoelectronic devices can lead to a significant weakening of the optoelectronic performance. To overcome this problem, Pei et al. introduced Ru-based dye complexes (organic components) for interface modification, which enhanced the chemical compatibility between heterogeneous materials and improved the photovoltaic conversion [138]. Liu et al. treated indium tin oxide film by supercritical carbon dioxide fluid which passivated the suspended bonds at the interface through the dehydration effect and introduced In^3+^ ions in the grain boundaries, resulting in an indium tin oxide-like structure at the grain boundaries, which modulated the interfacial defects in the photovoltaic film and improved the photoelectric conversion [139]. Surface plasma treatment also reduces oxygen vacancy defect states in materials such as gallium oxide and controls carrier transport, thereby improving photovoltaic performance [104]. Zeng et al. treated MoS_2_/graphene heterojunction FETs by swift heavy ion irradiation, which led to local annealing of graphene, thereby reducing the resistance and increasing carrier mobility, and the radiation-generated defects were able to induce increased light scattering, thereby modulating the photovoltaic conversion [105]. Inspired by dye-sensitised solar cells, the photovoltaic properties of devices can also be improved by modifying dye molecules (e.g. methylene blue, Rhodamine 6G, etc.) on the surface of the material or by complexing with them [119]. Yu et al. also optimized the performance of ZnO:Ga/SiC UV photodetectors by PtNP coating, by enhancing the photoelectric conversion efficiency, suppressing interfacial defects and capture centres, etc. [140].

The poor stability of tin-based perovskites limits their applications, which can be improved by modification or passivation through surface/interface engineering. Peng et al. improved the stability of tin-based perovskites by coating the surface with a 2DSnS mono-molecule layer. This interface allows for the flow of electrons and the formation of a built-in electric field, which has an impact on the optoelectronic performance; moreover, the surface SnS molecular layer also enhances the light absorption and inhibits iodide migration to improve the stability of the perovskites. Yue et al. proposed for the first time the modification of all-inorganic perovskites based on s-triazine and its derivatives. The stability of this optoelectronic device is improved by enhancing the intermolecular π–π interactions of the non-conjugated organic ligands, which improves the carrier transport and thus increases the photocurrent by a factor of 20, as shown in Fig. 15g, h [141]. It is worth noting that the type of substituents in conjugated organic ligands has an impact on the photoelectric effect, and better modifying substances should be explored in the future [141].

**Fig. 16:**
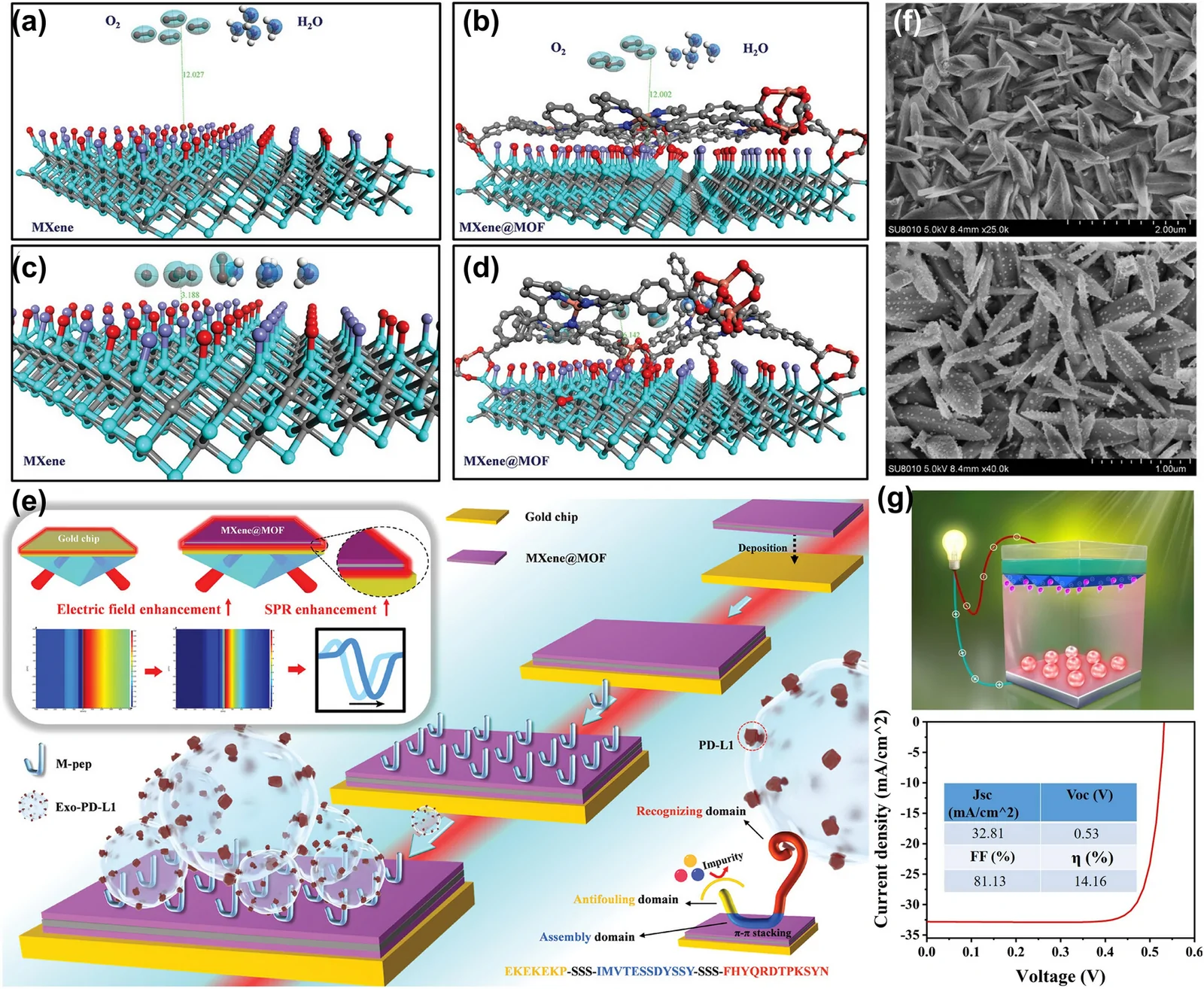
Optoelectronic performance optimization through local surface plasmon resonance. Optoelectronic performance optimization by Mxene@MOF-enhanced local surface plasmon resonance. **a**, **c** The easy oxidation characteristics of MXene. **b**, **d** The antioxidant properties of MXene@MOF. **e** MXene@MOF-enhanced surface plasmon resonance photodetector structure [143], Copyright 2023, Small. **f** SEM of before (above) and after (below) Au nanoparticles enhanced local surface plasmon resonance for SnO_2_ tetragonal nanonails [91], Copyright 2024, Ceramics International. **g** Structure of thin-film TiO_2_/c-Si heterojunction for enhanced local surface plasmon resonance [152], Copyright 2021, Results in Physics

MXene, as a 2D conductive material, has demonstrated its applicability in photoelectric conversion; however, the disadvantage of its high oxidation susceptibility limits its application and requires surface/interface engineering for modification [142]. Wang et al. formed a heterojunction by in situ growth of a 2D metal–organic framework on the surface of MXene, which not only inhibits the oxidation of MXene but also optimizes the photoelectric properties of MXene. It is also suitable for surface plasmon resonance sensitization requirements, which is an excellent optoelectronic interface material and shows great potential for bio-detection (Fig. 16a–e) [143].

Lead sulphide quantum dots are easy to handle in solution so are more convenient for their surface modification [144]. The energy conversion of quantum dot optoelectronic devices can be improved by introducing ligand exchange reactions in polar solvents [145]. In addition, in lead sulphide quantum dots, passivation through the introduction of hybrid iodide ions (I-) and the removal of organic ligands from the surface will enhance the light-to-dark ratio of the device, which will play a role in the optimization of photovoltaic effect [144]. Lead sulphide quantum dots are composed of a lead sulphide core and a lead shell, and through the above passivation process, the iodide ions replace the original long-chain organic ligands on the basis of the lead shell not being oxidized to reduce the spacing between the quantum dots, which enhances the carrier mobility. In addition, the replacement can also be carried out through other halide ions or through other non-long-chain organic ligands to reduce the polygon lifetime, enhance the mobility and enhance the electronic coupling, to improve the optoelectronic properties of the quantum dots [146, 147]. Zinc oxide quantum dots can also be prepared by solution, and it was shown that with the increase of annealing temperature, the crystal size increases, the specific surface area decreases, the responsivity decreases dramatically, and the response rate decreases, which is not favourable for photovoltaic conversion [148]. Shmid et al. investigated the improvement of the photovoltaic properties of germanium quantum dots on the surface of single-crystal silicon, which showed that the improvement of such photovoltaic properties is affected by the mechanical strain between the quantum dots and the silicon plane. The interfacial strain caused by the presence of the quantum dots enhances the charge separation distance and limits the recombination centres, thereby extending the lifetime of the photogenerated carriers [149].

Localised surface plasmon resonance is the process that electrons are gathered into regions of positive charge excess when a metal is subjected to electromagnetic interference, then oscillate back and forth at an equilibrium position under the influence of momentum, charge gravity, and inter-electron repulsion. Localised plasma resonance improves light absorption and helps to excite more energetic active electrons, enhancing the optical and photovoltaic performance of semiconductor optoelectronic devices [91]. The localised surface plasmon resonance of optoelectronic devices can be enhanced by introducing precious metals such as gold, silver, and platinum. After the introduction of gold nanoparticles, the photocurrent density of titanium dioxide nanowire arrays is enhanced by 16 times, the photocurrent density of zinc oxide nanorods is enhanced by 11 times [150, 151], and the photovoltaic performances of tin oxide tetragonal nanoparticles are also improved, as shown in Fig. 16f [91]. The grain boundaries of perovskites can also be lowered by introducing gold nanoparticles to enhance the film quality [76]. Wang et al. improved the photovoltaic performance by introducing metal nanowires to enhance the plasmonic resonance [2]. Krit et al. also used nanoclusters to enhance the localised plasmonic resonance to improve the photovoltaic performance [98]. Schottky junction optoelectronic devices facilitate the migration of photogenerated carriers, which can further amplify the amount of photogenerated electrons by combining with the local plasmon resonance to enhance the photoelectric conversion; however, this also simultaneously increases the transfer of hot electrons generated, which counteracts with the photogenerated carriers, thus weakening the photocurrent [98]. To address this challenge, Li et al. designed gold core silver shell/silver iodide Schottky junction contacts to stabilize the contacts by in situ growth. By adding a gold core, the localised plasmon resonance enhances the photogenerated electrons without increasing the generation of hot electrons, thus improving the photovoltaic performance dramatically [98]. Zhao et al. also enhanced the localised plasmon resonance by silver nanoparticles, which are confined around the nanometal. By designing a coupled structure of metal nanoparticles and titanium dioxide trigonal prisms, it can enhance electron transport and reduce carrier annihilation and thus improve the photoelectric performance; also, the presence of metal nanoparticles leads to light scattering and thus increases the light propagation distance to enhance the overall absorption, as shown in Fig. 16g [152]. Peng et al. probed the effect of gold, silver, and bismuth on the photoelectricity of 2D perovskites. Bi system has the lowest energy and the most stable adsorption; also, due to the electron orbital hybridisation, Bi has better carrier transfer and stronger adsorption, which implies a better surface polarisation effect and photovoltaic conversion [153]. Dong et al. also enhanced quantum efficiency of the photovoltaic devices through piezoelectric materials surface acoustic waves generation [154].

The polar structure of ZnO determines that it is more susceptible to spontaneous polarisation and has good piezoelectric properties, and research calculations have shown that polar ZnO (0001) has a smaller interlayer contraction distance than ZnO (000-1), with absorption peaks shifted from the visible region to the UV region, and a shift from an N-type to a P-type semiconductor [155]. By constructing and tuning the polar characteristics of zinc oxide, it is possible to modulate its optoelectronic properties, showing potential in photodetectors, especially real-time modulated optoelectronic devices.

Stress also has an impact on the optoelectronic properties of materials, especially flexible materials. Shi et al. prepared a widely applicable aluminium nitride film, which can be stably bonded to surfaces with a variety of morphologies, and produce piezoelectric photonics effects by bending stresses, which enhances the responsivity and response rate. It also has a tunable energy bandgap subject to the bending curvature of the substrate and has no fracture after cyclic bending, showing great promise in real-time tunable flexible photodetection [156].

**Fig. 17:**
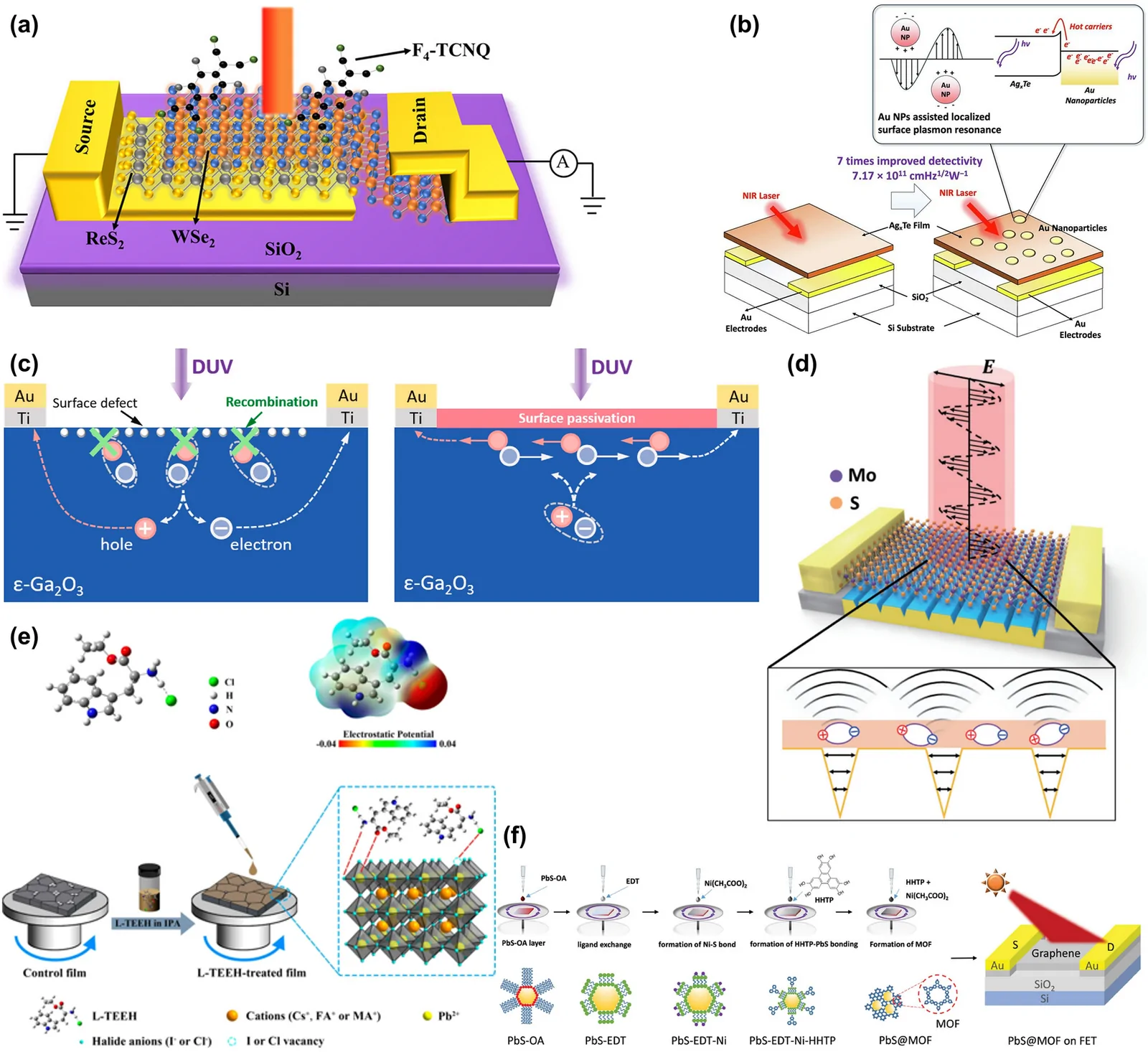
Various surface/interface modification schemes. **a** WSe_2_/ReS_2_ photodetector enhanced by surface charge transfer doping [160], Copyright 2023, ACS Applied Materials and Interfaces. **b** Ag_x_Te photodetector enhanced by Au nanoparticles induced localized surface-plasmon resonance [161], Copyright 2024, Applied Surface Science. **c** ε-Ga_2_O_3_ photodetectors enhanced by surface defect passivation [162], Copyright 2023, Materials Today Physics. **d** MoS_2_ photodetector enhanced by gap-surface-plasmon induced polarization photoresponse [163], Copyright 2023, Nano Research. **e** Lateral perovskite photodetectors enhanced by L-tryptophan derivative-induced surface passivation [164], Copyright 2023, Chemical Engineering Journal. **f** QD-MOF photodetectors enhanced by chemical bonding of interfacial engineering [165], Copyright 2023, Advanced Optical Materials

Smooth surfaces have good self-cleaning properties and repellency to a wide range of liquids, which is highly significant in device protection, including de-icing, corrosion protection, drag reduction, and fouling prevention [157]. Currently, this is mainly achieved by selecting suitable materials, designing surface microstructures, hydrophobic coatings, chemical solvent treatment and so on [157–159]. Some other surface/interface modification schemes are shown in Fig. 17.

### Design of Micro-/Nanostructures

#### Two-dimensional Heterostructures

Two-dimensional heterostructures arise from 2D materials, which can combine different strengths, provide functional complementarity, be easily integrated, and compensate for the disadvantages of a single 2D material [18]. Electrostatic interactions of 2D heterostructures can change the energy band structure of the material, and the built-in electric field at the interface can promote carrier separation and reduce unfavourable annihilation, providing higher charge transfer, which cannot be obtained by doping [166–168]. In addition, there are also sandwich-structured detectors, such as graphene-encapsulated TMDCs [169–171], which generate separated asymmetric potentials at the heterogeneous interfaces and enhance the response speed. Si et al. enhanced the charge transfer by improving the carrier lifetimes, which in turn promotes the photocurrent intensity by preparing van der Waals' two-dimensional heterogeneous MoS_2_/WSe_2_ photoelectrodes [117]. Li et al. prepared transverse TMDs heterojunctions with sharp interfaces and stepwise interfaces, respectively, and the comparison revealed that the sharp interfaces have better photovoltaic properties [106]. Shi et al. prepared nanorods with ZnO/BiOI heterostructures and constituted p-n heterojunctions to form an internal electric field, which promotes the electron migration and enhances the photovoltaic conversion efficiency [106]. Li et al. implemented in-sensor computing through interface engineering based on a heterojunction UV photodetector, which achieves edge detection and part recognition by constructing a single-layer neural network sensing array without the need for extra computational and storage components [172].

**Fig. 18:**
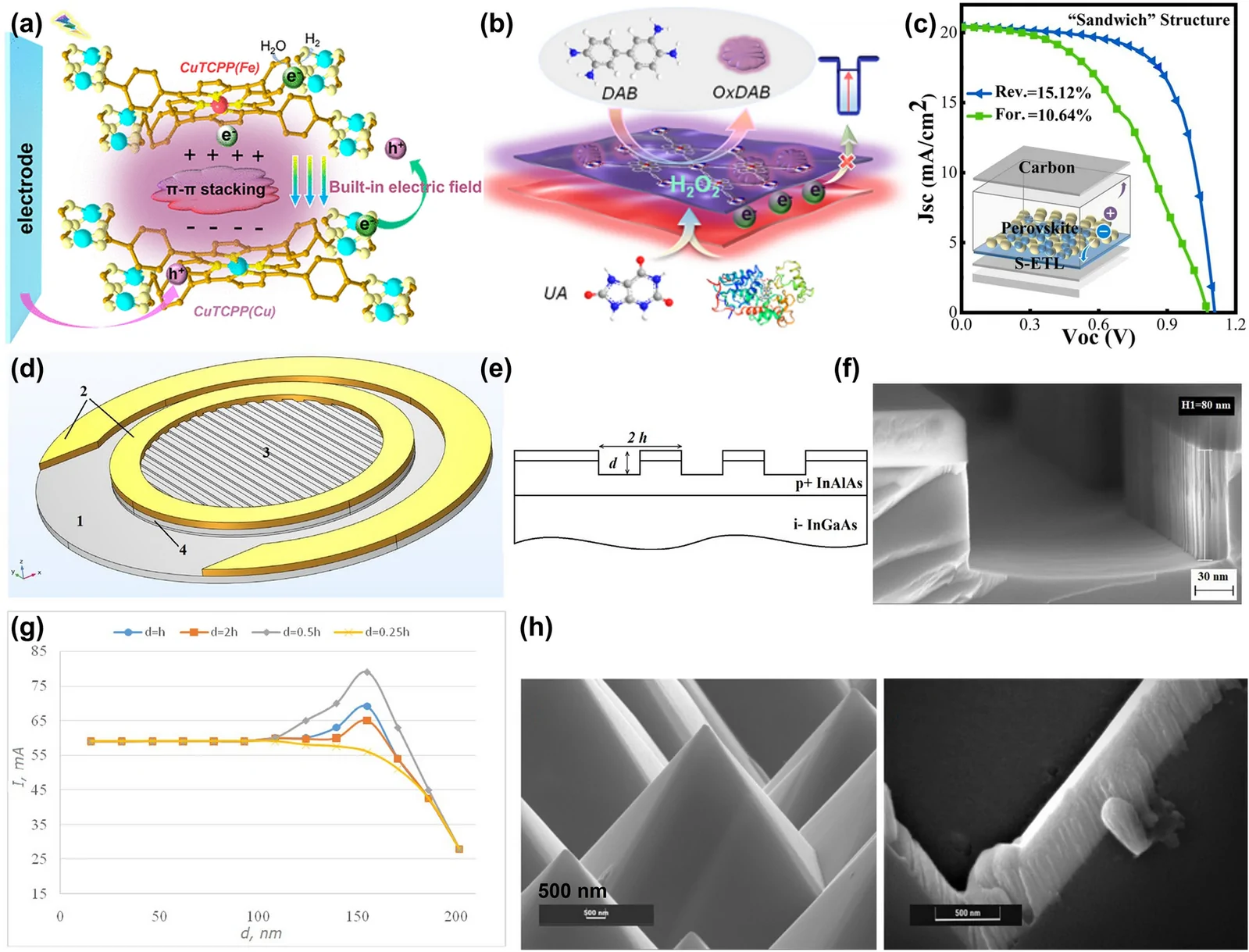
Optoelectronic performance optimization through micro-/nanostructure design. **a**, **b** Optoelectronic performance optimization by engineering the interfacial structure of heterojunctions [120], Copyright 2023, Nano Letters. **c** Carbon-based perovskite solar cell performance optimization by sandwich-structured electronic layers [173], Copyright 2023, Colloids and Surfaces A: Physicochemical and Engineering Aspects. **d–g** Optoelectronic performance influenced by the size of the ordered parallel groove [177], Copyright 2022, 28th International Symposium on Atmospheric and Ocean Optics: Atmospheric Physics. **h** Si/Sb_2_Se_3_ photodetector enhanced by micropyramidal structure [178], Copyright 2023, ACS Applied Materials & Interfaces

Liu et al. also generated a large built-in electric field by setting a bimetallic modulation centre on the heterojunction, which can effectively promote carrier separation and drive migration. The nanosheets of this heterojunction also reduce the migration distance and promote interface matching through π–π coupling, which reduces the interface out of carrier complexation, thus dramatically improving the performance of the photodetector and showing feasibility in biosensing, as shown in Fig. 18a, b [120].

Ye et al. prepared an electron transport layer for SnO_2_/TiO_2_/SnO_2_ carbon-based perovskite cells with a sandwich structure, where the mesoporous structure and interlayer structure enhance the excitation and transfer of electrons, which improves the photocurrent and the conversion efficiency, as shown in Fig. 18c [173]. Kang et al. also enhanced the electron transport layer of the carbon-based perovskite cells by inserting a titanium dioxide buffer layer between the VO_2_ and the glass substrate, which allows the titanium ions to diffuse and thus lead to a phase shift in VO_2_, resulting in a change in electrical resistance, which has an impact on the photovoltaic properties of the material [102]. Wang et al. enhanced the functional connectivity between the substances by inserting a graphene buffer layer at the interface of silicon and ZnO, which led to an increase in the electrical conductivity, and thus to an improvement in the photovoltaic utilization of the heterojunction [174].

#### Quantum Well Photodetectors

Quantum well photodetectors are realised by building a number of quantum wells into the light absorption layer of the detector, where leaps are generated inside the wells and are highly tunable as affected by the width and height of the wells [17]. Extending the carrier lifetime by designing shallow trap energy levels (i.e. the photogating effect) can enhance the responsivity of the photodetector [37].

#### Surface Texture Design

The surface texture design also has an effect on the optoelectronic performance, which is due to the correlation between the microscopic texture size and the wavelength of light [175]. Silicon is a typical optoelectronic material; it is important to study the effect of the microtexture of its surface on the optoelectronic performance. The surface texture also has an effect on the mechanical properties of optoelectronic devices. Considering the practical applications, the optoelectronic performance and mechanical properties should be considered in concert. Shpeizman et al. designed four texture structures on the silicon surface, including alkali solution selective etching of the surface, pyramidal texture, oxidation under the vanadium pentoxide layer and high-temperature annealing followed by hydrofluoric acid treatment. It was shown that the pyramidal texture has the worst strength and that the alkali solution selective etching is relatively the best [176]. Kulinich et al. investigated the effect of different surface textures on the photoelectric properties of InGaAs/InAlAs diodes, and it was found that on the surface of the ordered parallel groove diode, either too deep or too shallow grooves are not beneficial to the photocurrent generation, and the optimal depth is shown in Fig. 18d–g, and a reasonable shape and size should be designed for practical applications [177]. An ordered surface structure may also be beneficial to the improvement of photoelectric performance, as a uniform material distribution can reduce the film resistance and have good orientation and better stability. Han et al. prepared functionalized carbon nanotube photoelectric coatings with good ordering by the Langmuir–Blodgett method, which presented lower impedance and improvement of photoelectric performance [119]. Singh et al. set up a microcone structure on the silicon surface, and the responsivity of the device was enhanced nearly two times, as shown in Fig. 18h [178].

#### Low-dimensional Structure-Enhanced Photodetectors

One-dimensional structures such as nanotubes, nanowires, nanorods, and nanonails are favourable for the directional transport of carriers and can enhance the carrier migration rate and avoid compounding [91, 179, 180]. Nano-needle arrays perpendicular to the substrate are able to enhance the effective detection area, reduce reflectivity, and provide directional fast transport routes for photogenerated carriers, which is conducive to enhancing photovoltaic conversion [134]. Jiang et al. designed ZnO nanowire/molybdenum disulphide nanosheets, which show great potential in photodetection [181]. Lv et al. designed and prepared periodic CsPbCl_3_/CsPbI_3_ nanowire superlattices, forming one-dimensional heterostructures with uniform lattice orientation. It has higher responsivity, detectivity, and I_ON_/I_OFF_ ratio than non heterostructures [182]. Guan et al. designed a single-crystal PN junction perovskite nanowire array to enhance carrier transport through single crystals and effective carrier separation through PN junctions, thereby improving the sensitivity of photodetectors [183].

2D materials also have excellent performance in light detection. Tao et al. prepared 2D independent germanium-based monosulfide compounds nanoplates, which can achieve adjustable optical bandgap and electronic band through low-pressure rapid physical vapour deposition, and have great potential in the field of controllable polarization optics [184].

The formation of heterostructures by combining 1D nanomaterials such as nanowires with other 2D or bulk materials is also a way to prepare high-performance photovoltaic materials. Zhao et al. systematically summarised an effective method to precisely manipulate the growth of ZnO nanowires on other matrices through interfacial engineering techniques, whereby the carrier transport behaviours and energy-band structures of the ZnO nanowires are tuned to be altered and applied to different situations [100]. In addition to the traditional interfacial engineering such as doping, shape modification, and strain control, the study of overcoming the dangling bonds and defective states of 1D materials and mitigating phenomena such as interfacial mismatch is also an important part of 1D photovoltaic materials. Moreover, due to the high cost and reagent waste of the traditional epitaxial construction method of heterojunctions, the growth method based on van der Waals interactions shows the advantages of cost and easy fabrication, and the construction of heterojunctions with the help of self-assembly and so on is very promising in optimising the optoelectronic properties [100]. Liu et al. have also prepared microfloral BaTiO_3_@TiO_2_ composite piezoelectric photovoltaic materials with a heterogeneous interface, which can promote charge separation [185].

#### Functional Structure Enhanced Photodetectors

**Fig. 19:**
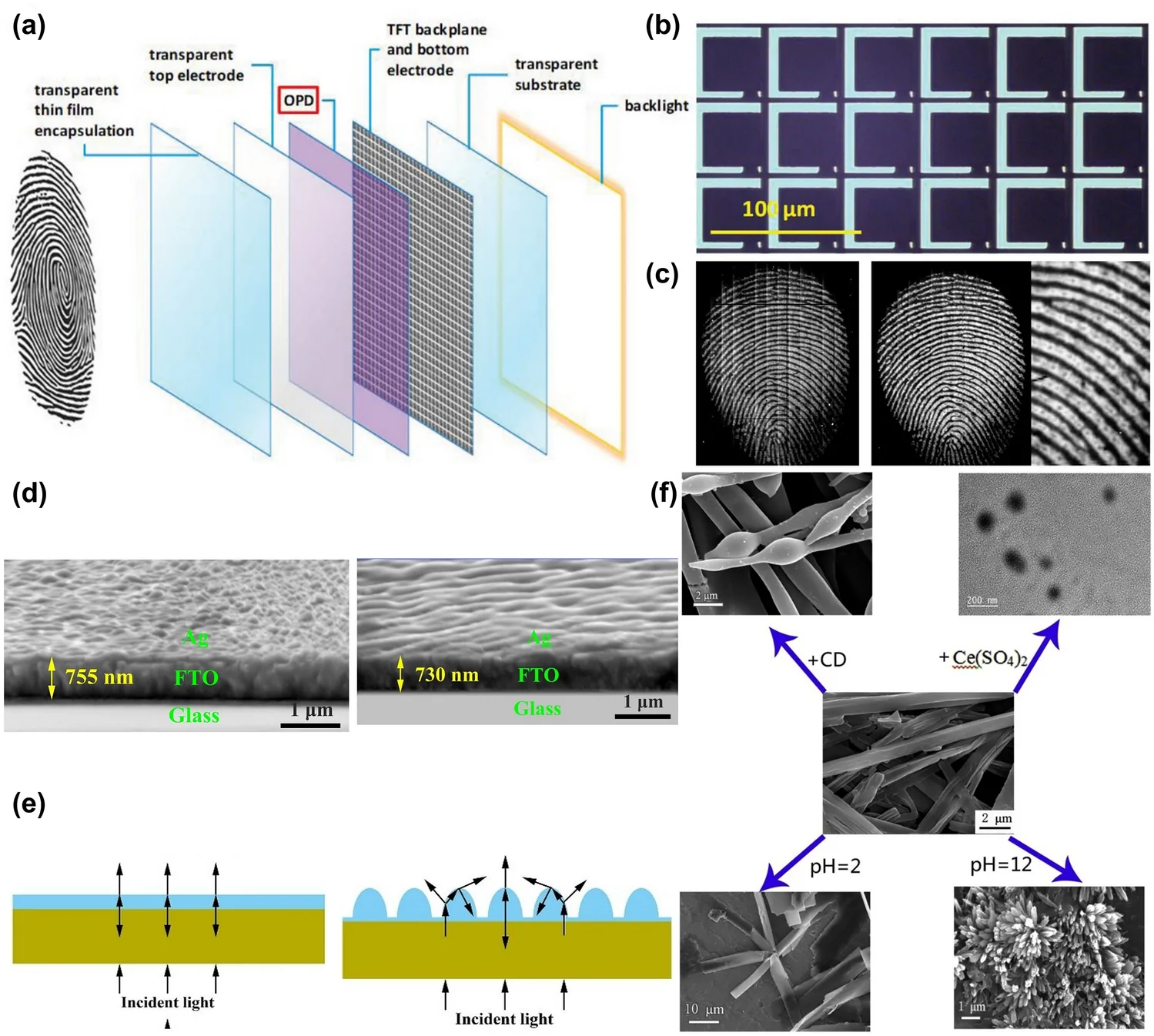
Optoelectronic performance optimization through micro-/nanostructure design. **a, b** High-resolution integrated optoelectronic fingerprint detection [186], Copyright 2019, Advanced Materials Technologies. **d**, **e** Ag/FTO optoelectronic performance optimization by antireflection grating structures [189], Copyright 2019, Applied Surface Science. **f** Optoelectronic performance optimization by functional hierarchical structures induced by different substances/pH [123], Copyright 2019, Colloids and Surfaces A

The performance can also be optimised by designing the functional structure of the detector. Tordera et al. formed many heterojunctions on a fingerprint sensor by slot-mode coating to make a large-area microdetector integrated array for high-resolution fingerprint recognition, as shown in Fig. 19a–c [186]. Its bottom gate can not only be used to increase the transistor current, but also provide light shielding to improve the device stability. Xu et al. also optimised the detector by designing a bowtie antenna and an aperture plasma structure, which improves the polarisation selection of the photodetector and light absorption [187]. The performance of the photodetector can also be improved by increasing the bottom gate configuration, due to the fact that the bottom gate imposes a large tunnel barrier at ungated channel regions, which facilitates the lowering of the free-electron barrier, so the photocurrent is improved while the dark current is suppressed [188]. Surface materials with high transmittance cause significant light loss and reduce light absorption, which is detrimental to the photoelectric conversion efficiency. Extending the light propagation path distance by designing absorbing structures or multiple reflections can help to improve the absorption. Wang et al. generated periodic, uniform reflective gratings on photovoltaic thin films by ultrasonic vibration-assisted laser irradiation, with a concomitant annealing effect that reduces the transmission loss, and enhanced local plasmon resonance with the aid of microstructures, which improves the photovoltaic properties of the materials, as shown in Fig. 19d, e [189]. Borodaenko et al. formed periodic nanogratings on PN junction photodetectors with high polarisation sensitivity to incident radiation by laser light, substantially increasing the effective area for light absorption [190]. Some semiconductor materials have broad absorption, which is very unfavourable for specific wavelength detection, so require the use of triple primary colour filter arrays to improve the optical intensity [1]. Optical gain can also be effective in improving signal strength [37].

#### Resonant Cavity-Enhanced Photodetectors

If the thickness of the detector is too small, the light is not completely absorbed, a reflective layer can be added to the backside so that the reflected light is further absorbed, thus enhancing the incident photon absorption, and this type of photodetector is known as resonant cavity-enhanced photodetectors. If a reflective layer is also provided on the surface of the absorber, the light will be reflected in the cavity between the surface and backside, thus enhancing the absorption, especially the resonant absorption [17]. Photodetectors with pin structure perform the function at the end of the fibre optic, which is able to process optical information and RF signals and plays an important role in remote communication [18]. These kinds of Schottky junctions or diode detectors are able to shorten the working length by regulating the photons highly coherent and directional through the resonant cavity and are able to modulate the incident light to achieve tunable bandwidth and high sensitivity [191]. The attenuation of efficiency can be improved by Fabry–Perot-type resonant geometry [192].

Photodiodes are based on the absorption of photons larger than the energy generation gap (i.e. absorption); lasers are based on the excitation of electrons to higher substable energy levels and their release to the initial energy level to produce laser light (i.e. stimulated emission). They are based on different principles, so it is usually not possible to realise the common function of both through the same device [18]. However, a vertical cavity surface-emitting laser (VCSEL) epitaxial structure is similar to a photodetector, which has the potential for photodetection enhancement. By combining a VCSEL with a photodiode, its quantum well active region can be activated in specific modes, enabling the integration of lasers and photodetectors, which is highly promising in areas such as microwave photonics technology [18]. Its quantum well active region operates in photovoltaic mode or reverse bias mode. It has great potential for high-speed optoelectronic switches, optoelectronic frequency conversion transceiver base stations, etc. [18, 193, 194].

#### Other Design of Micro-/Nanostructures for Photodetectors

Ma et al. prepared sea urchin-structured quaternary nanocrystalline alloys with multimetallic nanomaterials whose plasmon resonance on the surface could enhance carrier–hole electron separation and electron transport, and their multimetallic synergistic effect also enhances the photovoltaic performance [159]. Li et al. also improved the carrier-separation rate and shortened the transport distance of the bulk charge to the surface by a porous silver-oxide structure, with an improved photovoltaic performance [195]. Zhang et al. prepared clustered and dotted Bi_2_WO_6_-CdS hybrid nanocrystals, both with optimised optoelectronic properties [196]. The nanoferrite BiFeO_3_ undergoes a shape change (rhombic to cubic) by doping, which alters the magnetic and optoelectronic properties, and is an inspiration for the controllable preparation of photodetectors [197]. Sui et al. developed a photovoltaic material with a controllable hierarchical structure, which can be modulated by β-cyclodextrin inclusion, redox stimuli and PH changes, as shown in Fig. 19f. This is of great significance for photovoltaic molecular switching devices [123]. Liu et al. induced the spreading of SnS_2_ nanosheets and the change of hexagonal cadmium sulphide into rods through the glutathione guiding agent, which increased the light-absorbing area and photovoltaic conversion, and demonstrated its effectiveness in photocathode protection [114]. Nanostructures such as nanoparticles, gratings, and antennas can also enhance quantum efficiency [198–200].

The behaviour of electron–hole pairs at the interface has a great influence on the optoelectronic performance of the device, and much interfacial engineering has been carried out to modulate the mobile behaviour of electron–hole pairs by modifying the functional dots to achieve better optoelectronic performance [128]. Liao et al. designed photovoltaic material interfaces containing gold- and nitrogen-doped carbon dot components, and it was found that the gold could increase the electron–hole separation, while nitrogen-doped carbon dot can increase both the separation and the rate of carrier pairs, which is very favourable for the enhancement of photoelectric conversion.

**Fig. 20:**
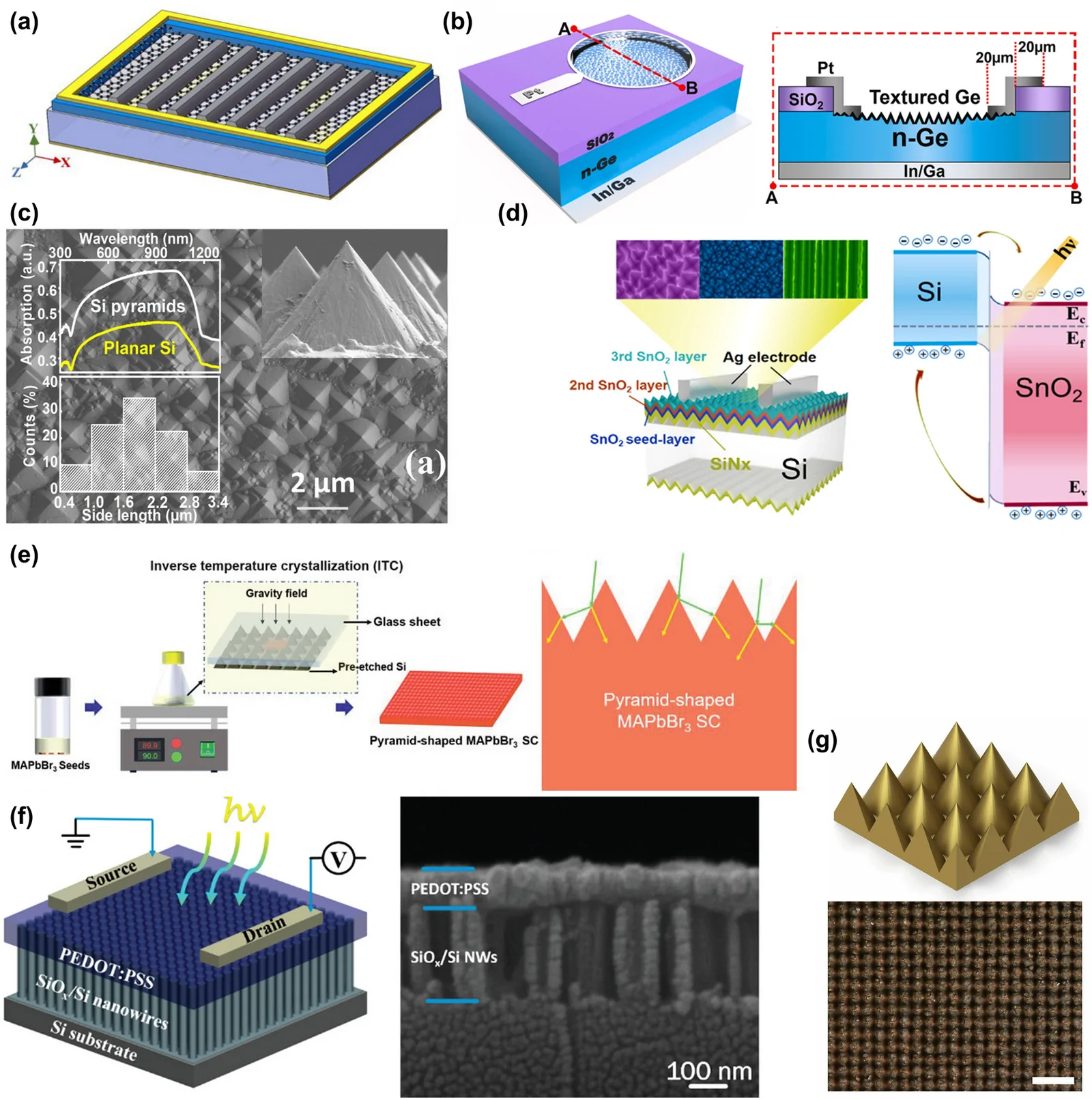
Various surface/interface structure design schemes. **a** Optoelectronic performance optimization by ellipse wall grating‑nanowire structure [2], Copyright 2019, Plasmonics. **b** Optoelectronic performance optimization by anti reflective surface texture [202], Copyright 2024, Materials Science in Semiconductor Processing. **c** Optoelectronic performance optimization by pyramid microstructure [203], Copyright 2024, IEEE ELECTRON DEVICE LETTERS. **d** Optoelectronic performance optimization by pyramid/V-groove [204], Copyright 2023, ACS Applied Materials & Interfaces. **e** Optoelectronic performance optimization by pyramid-shaped structures [205], Copyright 2023, Advanced Optical Materials. **f** Optoelectronic interface mediation by nanowires and inorganic/organic hybrid structure [206], Copyright 2023, Advanced Materials Interfaces. **g** Thermoelectric photodetector enhanced by periodic micro-taper array [207], Copyright 2024, Optics and Laser Technology

Reducing the thickness of the metal layer of the Schottky section also increases the responsivity [201]. Optimisation of various processes is also important for detector performance improvement. Enhancing the contact connection between the device circuits, choosing suitable contact point materials [86], and reducing the interface resistance are also important for device performance improvement. Photocurrent can be achieved by reducing the complexation of opposite carriers, e.g. reduction of defects, type ii band-aligned heterojunctions, etc. [18]. Some other surface/interface structure schemes are shown in Fig. 20.

Usually, surface/interface modification is achieved by using some functional groups or coatings to prevent material surface deterioration, which helps the detector maintain good performance and improve its stability. However, an excessively thick protective layer can block some of the incident light and reduce light absorption. Therefore, for the surface modification layer of the photosensitive absorber, it should be as thin as possible to minimize light loss while protecting the material. By increasing the specific surface area of the light absorption layer, the light absorption area can be significantly increased, which is beneficial for enhancing photoelectric conversion. However, this structure is also prone to carrier recombination and annihilation, so appropriate surface roughness should be designed. Reducing deep trap and enhancing surface light trap through defect passivation are also an important aspect. Deep trap is not conducive to carrier separation, while shallow trap can significantly improve the photoelectric conversion ratio. The design principle of surface quantum wells is similar, which enhances photoelectric conversion by improving surface light capture. Surface plasmon modification utilizes the plasmon resonance effect to capture photons more efficiently in the absorption layer. It can be excited with minimal thickness, while excessively thick coatings can hinder photons from entering the photosensitive layer. In optoelectronic devices, different parts may have mismatch due to different materials or crystal types, resulting in transmission losses, so interface modification is needed to reduce losses. In PN junctions or some designs, special heterostructures are required to promote carrier separation, generate potential differences in external circuits, and achieve photoelectric conversion. The design of surface patterns has diversity, which essentially increases the specific surface area to enhance light absorption. Some special patterns, such as planar antennas, can also achieve selective detection by selecting specific wavelengths. Low-dimensional photodetectors often have high performance due to their high uniformity; also, due to their small size, they are easier to prepare highly integrated optical sensing arrays, which have outstanding advantages in the fields of miniaturization and integrated circuits. Some functional structures/interfaces, such as absorption layers, multiple reflection layers, and resonant cavities, improve photoelectric conversion efficiency and optimize photodetectors by enhancing light absorption. These structural designs serve to assist absorption and reduce light loss, so excessive thickness should be avoided. In a photodetector, there are multiple surfaces/interfaces, such as photosensitive layer, photoelectric conversion layer, PN junction, and insulation layer. Different parts require different designs and modifications based on their functions; moreover, differences in materials and preparation processes can also lead to variations in the required interface engineering design. Micro-/nanostructure design and modification complement each other, exerting different functions by acting on different positions of materials/devices. In practical applications, interface engineering design should be carried out based on the specific requirements of the detector, and parameters should be adjusted according to the detector indicators.

Surface/interface engineering plays an important role in optimising the performance of optoelectronic devices and is the basis for the development of new optoelectronic devices. The usual means is to apply protective and passivation layers on the surface, which in turn protects the internal materials from deterioration and thus maintains a better photothermal performance. In addition, buffer layers, modified ions or functional groups can be inserted to alleviate mismatches between different materials. Laser surface treatment, plasma treatment, UV treatment, etc., can also modify the surface/interface to achieve the desired effect. Direct metal compositions are able to modulate and optimise the optoelectronic properties through local plasma resonance. However, these treatments are often limited to a particular material and are not widely applicable, and there is great potential for developing universal surface/interface modification solutions. The design of micro-/nanostructures is relatively less restrictive and is applicable to almost all types of materials. However, microstructure processing often requires highly sophisticated equipment for manipulation. Currently, there are still relatively few nanostructures designed for photovoltaics, and more functional microstructures need to be developed to enhance light absorption and energy conversion. Some surface/interface engineering for photodetectors in the past year are shown in Table 3. The influence of surface/interface engineering on photodetector parameters is shown in Table 4.

**Table 3:** Some surface/interface engineering for photodetectors in the past year

Number	Materials	Type	Method	Function	References
1	Si/Sb_2_Se_3_	Modification + Structure	Light-sensitive Sb2Se3 coating, Si micropyramidal substrate	Enhance absorption, excite surface-plasmon-polariton, harvest hot electrons	[178]
2	InAs/ZnSe/ InSb	Modification + Structure	InAs/ZnSe coating, chloride ions introduce, InSb quantum dot	Validate photoluminescence, improve photostability, prevention of oxidation	[208]
3	graphene/GaAs	Structure	Ellipse wall grating-nanowire	Surface plasmon resonance-enhanced carrier separation and transfer	[2]
4	ZnGa_2_O_4_	Modification	Surface fluorine plasma sensitization	Passive oxygen vacancy defects, change crystal grain boundaries	[209]
5	WSe_2_/ReS_2_	Modification	Surface charge transfer doping	Reduce Schottky barrier, increase the built-in electric field	[160]
6	α-Fe_2_O_3_	Modification	polyvinyl alcohol surface passivating	Surface defects suppression, increases carrier transport	[110]
7	Te/PbS	Modification + Structure	Te induced local surface plasmon resonance, PbS nanorod	Surface plasmon resonance-enhanced light absorption, excite the electrons conducted at interface	[96]
8	CsPbBr_3_/GaN	Modification + Structure	Surface-modified Ag nanoparticles, CsPbBr_3_ microwire	Passivation of surface defects, enhancing light absorption, improving photosensitivity, accelerating electronic transport, promote stability	[210]
9	Ag_x_Te	Modification	Plasmonic Au nanoparticles	Localized surface-plasmon resonance increased light absorption, enhanced hot-carrier injection	[161]
10	Diamond	Modification	Surface-modified indium nanoislands	Localized surface-plasmon resonance increased light absorption	[211]
11	Porous silicon	Modification	Surface-modified Ag nanoparticles	Reduce dark current	[212]
12	ε-Ga_2_O_3_	Modification	Al_2_O_3_ surface passivating	Reduced tin species and oxygen vacancies, enhanced surface carrier transport	[162]
13	Graphene/ ZnGa_2_O_4_/Ga_2_O_3_	Structure	ZnGa_2_O_4_/Ga_2_O_3_ core–shell structure nanowire networks	Improve the response time	[213]
14	TiO_2_/ BaTiO_3_	Modification + Structure	TiO_2_ core—BaTiO_3_ shell nanorods	Reduce carrier recombination and interface transfer resistance	[115]
15	AlN/GaN	Modification	Hexagonal close-packed structure coating with the individual quasi-triangular-shaped rhodium nanoparticle	Reduce dark current, improve responsivity	[214]
16	Silicon/ SiO_x_	Modification + Structure	Silicon Nanowire Length Control and SiO_x_ Surface Passivation	Improve optical absorption, induce the high photogain	[125]
17	Cs_4_PbBr_6_/PbSe	Modification	Halide ions (Cl − , Br − , or I −) passivating,	Improve interface defect states	[118]
18	Ge	Structure	Surface-textured Ge	Antireflective	[202]
19	MoS2	Structure	Plasmonic nanocavity	Enhanced light absorption	[163]
20	Si	Structure	Surface state and pronounced light confinement effect of pyramid microstructure	Improve wavelength selectivity and sensitivity	[203]
21	Triple-cation (Cs/FA/MA) mixed halide perovskite	Modification	L-tryptophan ethyl ester hydrochloride passivate the uncoordinated ions (Pb2 + and I-) and halide (I and Cl) vacancies	Reduce defects	[164]
22	SnO/ RhB	Modification + Structure	RhB dye molecules surface sensitization, SnO2 microwire	Improve interface electron transfer mechanism	[215]
23	CuPc/MAPbBr_3_	Modification + Structure	Pyramid-shaped structure on anMAPbBr_3_, small molecule copper (II) phthalocyanine Modification	Enhanced light absorption and stability	[205]
24	InAs	Modification	Surface plasmon nanostructures	The near-field localization effect of the plasma structure	[216]
25	MAPbBr_3_	Structure	Growth of polycrystals MAPbBr_3_ on the surface of single-crystal MAPbBr_3_	Enhancing surface current and accelerating charge transfer	[81]
26	TaSe_2_/WS_2_/TaSe_2_	Modification	Self-passivation of TaSe_2_	Ensure the stability, achieving a high responsivity	[136]
27	SnO_2_/Si	Structure	Inverted pyramid texture	Improve responsivity	[204]
28	ZnO/Pt/AlN	Modification + Structure	ZnO nanorods, Pt/AlN shell	Plasmonic enhanced UV emission, localized the optical field in the shell	[217]
29	AlGaN/GaN	Structure	Surface nanohole	Light trapping in nanoholes prolonged light path and enhanced absorption	[218]
30	TiO_2_/GaO_x_N_y_/Ag	Modification + Structure	The type-Ⅱ band structure of the metal/semiconductor/metal heterojunction, the plasmon resonance effect of Ag	Promote the separation of carriers, reduce the recombination rate	[219]
31	MAPbBr_3_	Structure	Inverted hemisphere array surface	Reduce the reflection losses	[220]
32	WSe_2_/Bi_2_Se_3_	Structure	Ordered interfacial structure Bi_2_Se_3_	Topological surface state reduce scattering at the interface	[221]
33	TiO_2_/ poly(vinylalcohol)	Modification	Poly(vinylalcohol) passivates oxygen vacancies	Reduce surface trap states and dark current	[222]
34	ITO/PQT-12-Ag NP/Al	Modification + Structure	Metal surface plasmon resonance, sandwich-structure	High responsivity, high detectivity	[223]
35	Si	Structure	Integrated photon-trapping surface structures	Higher photoabsorption	[224]
36	Cu	Structure	Femtosecond laser fabricate surface periodic micro-taper array	Sensitivity enhancement	[207]
37	PEDOT:PSS/SiO_x_/Si	Modification + Structure	SiO_x_/Si nanowires, PEDOT:PSS interface modification	Effective trapping of incident lights, creation of interfacial potentials raised the work function	[206]
38	MA_x_FA_1-x_PbI_3-y_Cl_y_	Modification	2-ethyl-1-hexylamine interface treatment	Improve the hydrophobicity, enhance the hole transport layer, optimize the nucleation and growth of crystals	[225]
39	ITO/perovskite	Modification	Introduction of m-MTDATA	Reduce of surface traps, facilitate the charge separation	[226]
40	SnS_2_/MXene	Modification + Structure	SnS_2_/Nb_2_C heterojunction, Parylene-C encapsulation	Preserve oxidation, enhance the charge transfer, carrier density, light adsorption	[227]
41	Au NP/PbS	Modification + Structure	Plasmonic Au NPs, PbS quantum dot	Achieve the direct interface connection of PbS and Au, improve light absorption	[124]
42	ITO/ ZnO	Modification	Dual Interface Modification Using Potassium Aspartic Acid	Improve the storage stability, suppress the defects	[228]
43	ITO/P_3_HT:PC_70_BM	Modification	Al_2_O_3_-modified PEDOT:PSS interfacial layer	Prevent the interfacial chemical reaction	[97]
44	PbS/MOF	Structure	PbS quantum dot and MOF hybrid composite	Enhance electronic conductivity and the chemical stability	[165]
45	TCNQ/copper thiocyanate	Modification	TCNQ doped copper thiocyanate anode interfacial layer	Enhance shunt resistance, increase depletion width, enhance hole extraction efficiency, reduce trap density of states	[229]
46	Perovskite	Modification	(4-hydroxyphenyl)phosphonic acid (OH) self-assembled monolayers	High charge transfer efficiency	[230]
47	Perovskite	Modification	CPTS self-assembled monolayers	Prolong excitons’ lifetime, produce a larger grain size	[231]
48	PTAA/PEDOT:PSS	Modification	Add small molecule DRCN5T	Passivate surficial defects	[232]
49	CsPbBr_3_/MoS_2_	Structure	Heterojunction	Effective separation of photogenerated carriers	[233]
50	CsPbIBr_2_	Modification	PbCl_2_ and Zn(DBDTC)_2_ for bottom and top interface modification	Passivate surface trap state, enhance hydrophobicity and resist phase segregation, improve interface contact	[234]

NP = nanoparticle; PC70BM = [6, 6]-phenyl-C70-butyric acid methylester; MOF = metal–organic framework; TCNQ = 7,7,8,8-tetracyanoquinodimethane; CPTS = (3-chloropropyl)trichlorosilane; DBDTC = Dibenzyldithiocarbamate

**Table 4:** Influence of surface/interface engineering on photodetector parameters

Number	Surface/interface engineering types	Parameter changes of photodetectors
1	Surface modification	Improve response uniformity, responsivity and detectivity, light absorption, and stability
2	Passivation	Improve response uniformity, responsivity and detectivity; light absorption, carrier separation, and response speed Suppress dark current
3	Surface plasmon resonance	Improve responsivity and detectivity, and light absorption;
4	Heterostructure	Improve responsivity and detectivity, carrier separation, and response speed Suppress dark current
5	Quantum well	Improve active area, responsivity and detectivity, light absorption, and carrier separation Suppress dark current
6	Surface texture design	Improve active area, responsivity & detectivity, and light absorption
7	Low-dimensional structure	Improve active area, and light absorption
8	Resonant cavity	Improve responsivity & detectivity, and light absorption
9	Antenna structure	Improve spectral sensitivity, responsivity & detectivity, and light absorption

## Applications

Photodetectors can have different signal outputs. Diodes, photomultipliers, transistors, photoresistors/thermistors, thermocouples, pyroelectrics, etc., output a single element, while pickup tubes, image intensifiers/converters, cameras, charge-coupled devices, focal plane arrays, etc., output an image [17]. Photodetectors are available in a wide range of types for different scenarios.

### Thermal Imaging

Thermal imaging based on heat detectors has a wide range of applications. It can take non-contact tests and verify the thermal performance of packaged systems such as printed circuits and power supply devices; it can also monitor mechanical systems, ionisation systems and detect malfunctions timely; it can assess heat dissipation and air circulation in enclosed/semi-enclosed spaces; it can also visualise aerodynamic interface interactions; so it plays an important role in diagnostics, troubleshooting and testing. In the medical field, thermal imaging can be used for early detection of breast cancer by comparing tissue activity and detecting circulatory abnormalities, showing great potential for non-contact diagnosis and treatment [235]. In thermal imaging design, surface/interface engineering significantly improves detection sensitivity by optimizing chip performance. It can identify small temperature changes and convert them into image signals. Surface/interface engineering improves thermal imaging performance by improving detection units and the interconnection between units. It can also reduce the size of detection units through the development of low-dimensional materials to enhance resolution.

### Monitoring

Near-infrared short-wave detectors have a wider range of applications, including communication, remote sensing, monitoring, identification, etc., due to the fact that near-infrared light (wavelength range: 1–3 mm) has higher penetration than visible light, higher energy than far-infrared, and is suitable for night-time applications [92]. Geum et al. achieved multicolour detection through a high-throughput epitaxial lift-off process. During the process, the visible light-sensitive GaAs were transferred to the NIR-sensitive InGaAs with the help of a Y_2_O_3_ bonding layer, and the two were vertically aligned to achieve multicolour detection [236]. Optoelectronic devices can convert optical information into electrical signals, which can be better processed by computer software. However, nonlinear optoelectronic conversion, analogue-to-digital conversion, noise, and interconnections can result in weakened or even failed effective information transfer, high energy consumption, low accuracy, etc. Chen et al. designed a fully analogue chip combining electronic and optical computation, which eliminates the need for analogue-to-digital conversion by directly encoding photocurrents and feeding them into integrated computational analogue circuits, with the advantages of low energy consumption and high computational speed, which is of great importance in the fields of wearable wearable devices, autonomous driving, and industrial inspection with great potential [237]. Surface/interface engineering can optimize the performance of monitoring equipment to meet the needs of medical, mechanical, aerospace and other fields. Moreover, surface/interface engineering will further promote the miniaturization and integration of monitoring chips, so enhance monitoring efficiency.

### Catalysis

The use of optoelectronic devices (such as semiconductor photoelectrodes, photoelectrocatalytic reactors, etc.) to combine light energy with electrical energy to achieve "photo-electrical-chemical energy" conversion can promote chemical reactions [238]. Compared with traditional photocatalytic reactors, photoelectrocatalytic reactors are easier to recover and reuse photocatalysts, and the efficiency of catalytic reactions is higher. For example, in the field of water splitting hydrogen production, the use of sunlight to split water to produce hydrogen in photoelectrochemical cell systems. This method converts unstored photocurrent into stored energy—hydrogen, which is considered the best hydrogen production method. Surface/interface engineering can increase the contact area of reactions and enhance reaction rates by high specific surface area, thus enhancing catalytic efficiency [239].

### Flexible Equipment

**Fig. 21:**
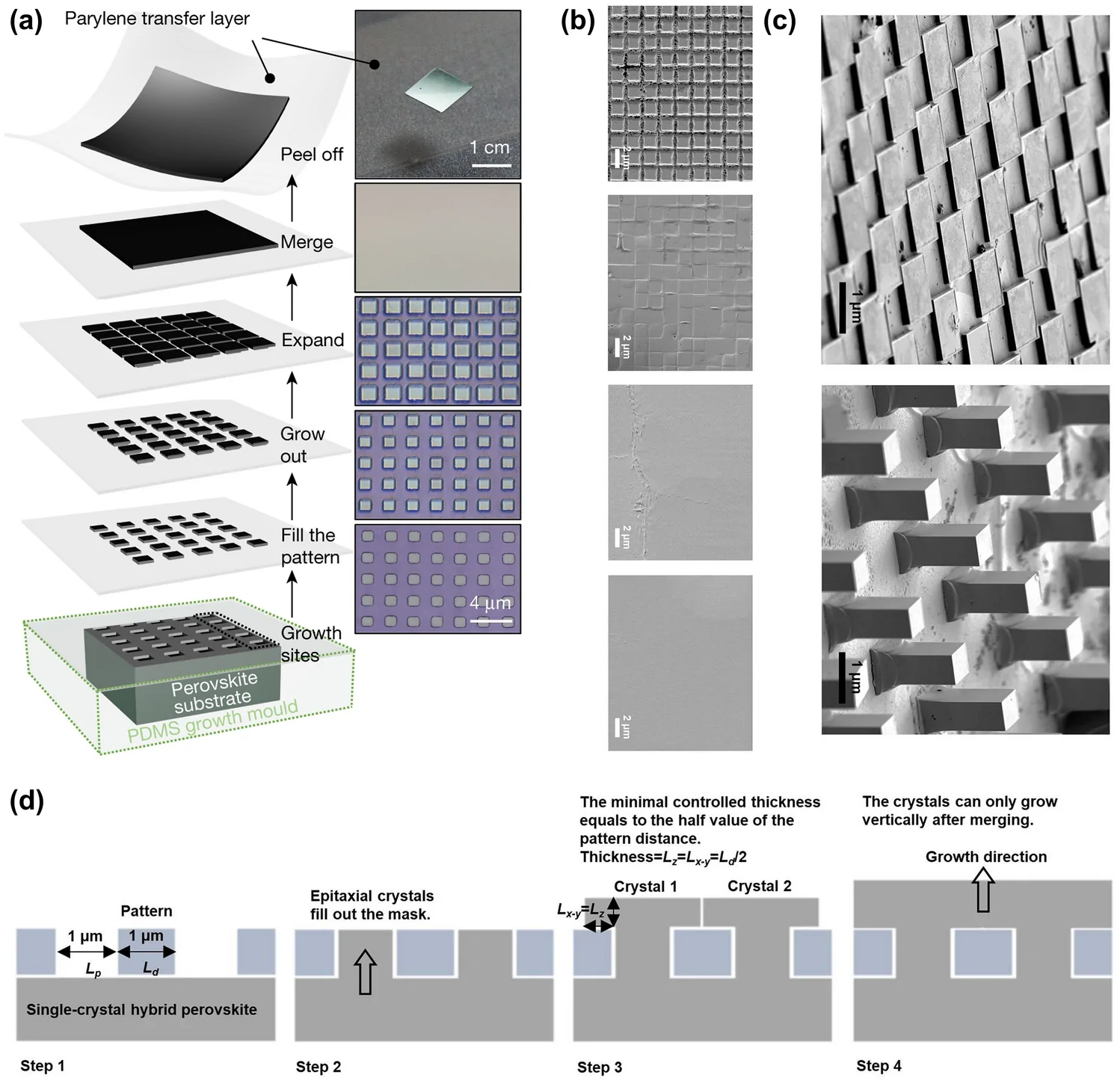
Flexible applications of single-crystal perovskite photodetectors [14], Copyright 2020, Nature. **a** Single-crystal perovskite thin film obtained by solution-based epitaxial growth. **b** SEM of epitaxial merging steps. **c** SEM of different growth temperatures and concentrations (above: low temperatures and concentrations; below: high temperatures and concentrations). **d** Epitaxial growth steps

The development of flexible photodetectors with variable shapes is highly promising with the development of wearable electronic devices, digital medicine, flexible medical facilities, soft robotics, etc. Organic materials are an effective means of realising flexible photodetection, and currently, narrow bandgap polymers can be prepared by various ways [240]. Donor–acceptor structures with narrower energy levels are formed by polymerising acceptors and donors to achieve 2 new highest occupied molecular orbital energy levels and 2 new lowest unoccupied molecular orbital energy levels [92]. Breaking the aromatic unitary structure and forming double bonds to develop the quinone structure also favours the formation of a narrower band gap [92]. Similar to inorganic semiconductor materials, polymer photodetectors are often designed with heterogeneous structures to achieve charge separation, usually with low bandgap polymers as donors and fullerene derivatives as acceptors, and doping of the donors and acceptors promotes charge-transfer absorption and extends the absorption edge [92, 241, 242]. The increase in interfacial area also favours charge-transfer absorption and is influenced by the thickness [241]. By preparing heterogeneous mixtures using phase separation, Gong et al. prepared a continuous inter-transfer donor–acceptor network, which greatly enhanced the sensitivity of the polymer photodetector and the spectral range covered a wide interval from the UV to the NIR [242]. In addition to polymers, organic small molecules have also been used to realise the photodetection function of electronic devices, with well-defined structures, easy batch preparation and high carrier mobility [92]. However, the charge transfer in this kind of substance is extremely weak and difficult to use for light detection, which can also be improved by designing microcavity structures [243, 244]. Typically, flexible light detection devices are based on bendable and malleable organic materials such as polymers. These materials are simple to prepare, do not require high temperatures and pressures, can be prepared at low cost over large areas, and have excellent flexibility, some of which are stretchable, making them valuable in the preparation of shape-variable thin-film detector devices [92]. Flexible optoelectronic devices are usually based on polycrystalline optoelectronic materials, single-crystalline materials have better optoelectronic properties compared to them due to higher orientation, low defect concentration, carrier transport stability, etc. The preparation of single-crystalline optoelectronic materials, especially their flexible realisation, has always been a great challenge [14]. Lei et al. achieved the preparation of single-crystalline perovskites on an arbitrary substrate by lithography-assisted epitaxy and transfer, as shown in Fig. 21 [14]. Currently, bio-interface photodetectors based on surface interface engineering are a key component in fields such as artificial intelligence and human–computer interfaces. Wang et al. comprehensively explored high-performance flexible silicon for photovoltaics, photodetectors, and bio-interfaces, as well as its morphology and surface interface engineering [245]. In fact, the development of flexible photodetectors cannot be separated from surface/interface engineering. Optoelectronic materials are usually inorganic, so it is necessary to study their adhesion and electrical characteristics with flexible substrates by surface/interface engineering. Moreover, flexible materials face interface strain caused by bending, so surface/interface engineering are essential for flexible equipment.

### Biochemical Sensing

Visual impairment is a disease that is difficult to overcome because visual damage is usually irreversible. Many researchers are exploring the possibility of mimicking the visual function of the human eye with the help of implantable photodetector systems to achieve retinal features and functions (0.1 visual cells per square micron) with the help of high-resolution colour recognition units [246]. Implanting photodetectors under the retina also has the potential for visual restoration, providing a viable option for the improvement and treatment of blindness and weak vision [247]. Gu et al. mimicked the function of the retinal photoreceptors of the human eye by means of a perovskite photovoltaic nanowire array, with the aid of gas-phase deposition to make the nanowires perpendicular to the surface of a hemispherical curved surface, and linking the front-end lens imaging to the back-end artificial liquid-metal nerve fibres, which converted the optical information into electronic information and display it on a computer, forming an artificial eye with the function of the human eye [246]. Since both the nanowire light-sensing units and the liquid-metal nerve units are independent of each other, this ensures higher information transfer speed and lower interference; the extremely small cross-sectional area of the nanowire allows for more independent units in the hemisphere, which is conducive to improving the resolution of the artificial eye. The surface/interface engineering of biochemical sensing is inspired by biological interfaces and biological microstructures. Materials inspired by gecko foot suction cups can have higher interfacial adhesion; devices inspired by the microstructure of lotus leaf surfaces exhibit better hydrophobicity. Surface/interface engineering is inspired by natural organisms, and through imitation, optoelectronic devices have functions comparable to or even surpassing the human eye.

Photodetectors also have several applications in energy conversion. In recent years, with the development and innovation of low-cost solar cell preparation technology, photodetectors also play an important role in the field of photovoltaics, especially low-cost photovoltaic innovative materials such as perovskite and copper indium gallium diselenide (CuInGaSe_2_). Photodetectors are also used in image printing and copying, where image reproduction is achieved by means of large-area photoconductors.

Photodetectors can also be used for photocathode protection. Liu et al. designed and prepared SnS_2_ nanoflake/CdS nanorod heterojunction photoanodes for the protection of 304 stainless steel [114]. Shi et al. also realised stainless-steel cathodic protection through ZnO/BiOI p-n nanorod photoanodes [103].

At present, the surface/interface engineering of micro-/nano-photodetectors has wide applications. Surface modification can effectively ensure the stable operation of photodetectors in certain environments, such as high humidity and high oxygen, so play a protective role. Moreover, interface engineering can optimize light absorption and improve the performance of light detectors. Interface engineering mainly involves microstructure design and modification to compensate for material defects and optimize the performance of photodetectors. Through surface/interface engineering, almost any existing photodetector can be optimized at the nanometre/micrometre level, making it suitable for a wide range of fields. For traditional semiconductor photodetectors, surface/interface engineering significantly reduces material defects caused by preparation processes; for low-dimensional photodetectors, surface/interface engineering not only optimizes device performance, but also provides guidance for low-dimensional device design through various microscopic effects, promoting the development of novel low-dimensional photodetectors. In the field of manufacturing, surface/interface engineering requires finer microstructures and controllable processes, which can promote continuous innovation in preparation techniques. Low-dimensional photodetectors are easy to design arrays so have great potential in the field of microchip based high-sensitivity photodetection. Inorganic or low-dimensional optoelectronic materials can be combined with flexible substrates for flexible devices, which have great potential in wearable devices. By applying hydrophobic surface modification, the photodetector can operate stably in aquatic environments and is suitable for underwater detection. In the field of imaging, low-dimensional high-array large-scale photodetectors are beneficial for improving image resolution. In summary, surface/interface engineering is committed to optimizing the performance of photodetectors through micro-modification and structural design, and is suitable for improving the performance of photodetectors in almost all applications. However, for specific application areas such as imaging, wearables, and monitoring, surface/interface engineering can only assist in the design of light detection functions, thereby optimizing detection performance.

Photodetectors have a wide range of applications in different fields due to their unique visual and digital interaction capabilities, which can be seen in many fields such as aerospace, remote sensing, maritime, biological, mechanical, and environmental. However, the cost of high-resolution photovoltaic devices, especially micro-sized direct photovoltaic conversion, is still a great challenge. The photovoltaic devices generate DC, which also limits their applicability. In the future, with the development of artificial intelligence and computer technology, high-resolution micro-/nano-optoelectronic devices will play a more important role.

## Summary and Outlook

In conclusion, this paper systematically introduces the principles, types, and parameters of high-resolution optoelectronic devices, and explains the scope of application of different types of optoelectronic devices. Afterwards, the advantages of various types of optoelectronic materials, especially 2D materials, for light detection applications are described. Different thin-film preparation processes and their post-processing methods are described in detail, aiming at reducing defects and optimising the structure to enhance the optoelectronic performance. In addition, we also give solutions to optimise the performance of photodetectors by surface/interface modification and micro-/nanostructure design, and hint at the potential for the development of innovative structured photodetectors. Finally, we show the wide range of applications of photodetectors and their future potential in human–computer interaction.

Based on the principle of photoelectric conversion, the light-sensing frequency interval of a material strongly depends on the gap of the substance. The band gap can be tuned by designing material combinations, and thus, we can expand the absorption band of the photodetector. In addition to direct photoelectric conversion devices, photothermal/thermoelectric devices are also commonly used light detection strategies, which have lower cost and optical frequency limitations and high conversion efficiencies, showing great potential in the infrared band. Novel materials such as piezoelectric materials and pyroelectric materials also have unique electrical effects that modulate the electron distribution and flow in the device. Thus, it is also very important to continuously explore new materials and new optical or electrical effects for the optimisation and innovation of optoelectronic devices. High-resolution micro-/nano-optoelectronic devices can improve the shortcomings of traditional devices by constantly discovering new effects to design novel optoelectronic devices. Moreover, the establishment of standardised parameter indicators is also very important for the lateral comparison and upgrading of optoelectronic devices, but the standardisation of optoelectronic indicators for devices of different types and sizes is still a long way from being achieved.

With the development of 2D, 1D, and 0D materials, the size of micro-/nanophotodetectors is reducing, which is favourable for resolution improvement. Scientists are also exploring new, low-cost families of optoelectronic materials, such as perovskite. With the development of wearable devices, flexible organic optoelectronic materials are also gradually entering the public eye and attracting attention. The preparation processes are also evolving, and to adapt to flexible substrates, reduce the preparation cost, and build unique heterostructures without affecting the photovoltaic performance, milder and faster preparation schemes are replacing the traditional processes, such as hydrothermal methods, self-assembly, etc. However, these methods are affected by electrostatic interactions, interfacial tension, capillary forces, etc., in the preparation of small-sized nodes, and lack of stability. Moreover, post-treatment processes can also be valuable in remedying defects and optimising materials for the improvement of optoelectronic devices.

Surface/interface engineering of optoelectronic devices can be carried out during or after the preparation process, which mainly includes material modification and structural modification. Material modification protects and modulates the physical properties of the device by coating or branching special groups, functional groups, substances or particles, etc., to optimise light absorption, electron excitation, electron migration, carrier complexes, etc. Microstructural modifications have a wider range of applications, including gratings, resonant cavities, shallow traps, micro-antennas, and various shapes of 1D materials and nanorods, which can enhance light absorption, reduce losses, modulate carrier behaviour, etc., and thus improve photovoltaic conversion. By studying the lattice and potential wells of materials, innovative microstructures can be explored to compensate for lattice defects and optimise optoelectronic devices.

Currently, surface/interface engineering still faces challenges in terms of controllability, preparation, and cost. The thickness and density of micro modifications require precise control of reaction dose and time, and even small differences may cause failure. In addition, the implementation of microstructures often requires the assistance of micro-/nano-processing techniques, which require reliable manufacturing technologies. The interface engineering can greatly optimize the performance of the photodetector, but its cost also further increase. In applications, a balance between performance and cost is needed. Also, some low-cost technologies can achieve surface/interface optimization. For examples, some surface treatments only require simple immersion in lead solution; annealing/heat treatment/hot isostatic pressing can also significantly eliminate internal defects. Surface engineering and micro-/nano-manufacturing technology complement each other; on the one hand, the implementation of surface/interface engineering depends on the continuous development and maturity of micro-/nano-manufacturing technology; on the other hand, micro-/nanomanufacturing technology will promote innovative modification and structure design, thereby enhancing the development of surface/interface engineering. In the future, surface/interface engineering will develop in collaboration with micro-/nanomanufacturing technology. Moreover, with the development of integrated circuits and the industrialization of low-dimensional optoelectronic materials, surface/interface engineering will significantly improve the performance of optoelectronic chips and innovation of optoelectronic materials. Presently, machine learning neural networks can be used to construct mathematical–physical models for surface/interface engineering. Through industrial big data, models can be promoted, controllable surface/interface engineering design and preparation can be achieved, further promoting industrialization. Artificial intelligence-assisted technology can help better achieve literature management and data statistics.

Photodetectors have a wide range of applications in aerospace, military, marine, medical, and environmental fields due to their visual and digital interaction features and have shown great advantages in the field of green and sustainable photovoltaics. Moreover, in direct photovoltaic conversion devices, photogenerated electrons are quantised, so direct conversion of optical signals to digital signals is feasible, which will greatly shorten the previous signal transmission path, so dramatically increase the speed of computation. In the future, with the development of artificial intelligence and robotics, photodetectors can not only provide self-supplied energy, but also capture image information and convert it into signals that can be processed by computer software, thus constructing an environment–computer interaction bridge and realising machine vision functions.
